# A Novel Method and Its Application to Measuring Pathogen Decay in Bioaerosols from Patients with Respiratory Disease

**DOI:** 10.1371/journal.pone.0158763

**Published:** 2016-07-07

**Authors:** Graham R. Johnson, Luke D. Knibbs, Timothy J. Kidd, Claire E. Wainwright, Michelle E. Wood, Kay A. Ramsay, Scott C. Bell, Lidia Morawska

**Affiliations:** 1 International Laboratory for Air Quality and Health (ILAQH), Queensland University of Technology (QUT), Brisbane, Queensland, Australia; 2 School of Public Health, The University of Queensland, Herston, Queensland, Australia; 3 Child Health Research Centre, The University of Queensland, Herston, Queensland, Australia; 4 Centre for Experimental Medicine, Queen’s University, Belfast, Northern Ireland, United Kingdom; 5 School of Medicine, The University of Queensland, Herston, Queensland, Australia; 6 Department of Respiratory and Sleep Medicine, Royal Lady Cilento Children’s Hospital, South Brisbane, Queensland, Australia; 7 Lung Bacteria Research Group, QIMR Berghofer Medical Research Institute, Herston, Queensland, Australia; 8 Department of Thoracic Medicine, The Prince Charles Hospital, Chermside, Queensland, Australia; Mount Sinai School of Medicine, UNITED STATES

## Abstract

This work aimed to develop an *in vivo* approach for measuring the duration of human bioaerosol infectivity. To achieve this, techniques designed to target short-term and long-term bioaerosol aging, were combined in a tandem system and optimized for the collection of human respiratory bioaerosols, without contamination. To demonstrate the technique, cough aerosols were sampled from two persons with cystic fibrosis and chronic *Pseudomonas aeruginosa* infection. Measurements and cultures from aerosol ages of 10, 20, 40, 900 and 2700 seconds were used to determine the optimum droplet nucleus size for pathogen transport and the airborne bacterial biological decay. The droplet nuclei containing the greatest number of colony forming bacteria per unit volume of airborne sputum were between 1.5 and 2.6 μm. Larger nuclei of 3.9 μm, were more likely to produce a colony when impacted onto growth media, because the greater volume of sputum comprising the larger droplet nuclei, compensated for lower concentrations of bacteria within the sputum of larger nuclei. Although more likely to produce a colony, the larger droplet nuclei were small in number, and the greatest numbers of colonies were instead produced by nuclei from 1.5 to 5.7 μm. Very few colonies were produced by smaller droplet nuclei, despite their very large numbers. The concentration of viable bacteria within the dried sputum comprising the droplet nuclei exhibited an orderly dual decay over time with two distinct half-lives. Nuclei exhibiting a rapid biological decay process with a 10 second half-life were quickly exhausted, leaving only a subset characterized by a half-life of greater than 10 minutes. This finding implied that a subset of bacteria present in the aerosol was resistant to rapid biological decay and remained viable in room air long enough to represent an airborne infection risk.

## Introduction

Controlled experiments on the aging of airborne viruses and bacteria can contribute much to our understanding of airborne respiratory infection transmission. However, such research has to date focussed mainly on laboratory-generated aerosols, which can differ from natural respiratory aerosols in their composition and mechanisms of production. There are several reasons for these differences. Firstly, the viability and infectivity of airborne bacteria and viruses depend strongly on the composition of the nebuliser suspension used to generate the aerosol and on the propagation techniques used to produce the aerosolised infectious agents[1–5]. Secondly, the aerosol composition that a laboratory experiment aims to replicate will depend on the specific location of aerosol production in the respiratory tract[6–9]. Finally, respiratory tract infections alter the respiratory fluid composition[6] and probably change rates of aerosol production[10]. Accurate replication of natural respiratory aerosol production and composition is therefore necessary in aerosol aging studies, but very difficult to substantiate because the processes underlying natural bioaerosol production are not well defined.

Accurate control of aerosol aging is essential when studying the decay of airborne pathogen infectivity. However, achieving this is complicated by the fact that the aerosol aging timeframes over which bacteria and viruses lose their initial infectivity can be very broad[11]. A wide range of aging intervals therefore needs to be considered.

Immediately upon exiting a subject’s respiratory tract, respiratory aerosols undergo rapid desiccation and cooling[12, 13]. A lower limit for the aging intervals to be investigated is therefore set by the evaporation time required for the respiratory droplet to form a stable droplet nucleus. Airborne respiratory droplet nuclei (DN) are usually assumed to be those with a diameter of less than 5 μm[14, 15]. For the liquid respiratory fluid droplets that produce such DN, the evaporation process achieves a stable equilibrium diameter within seconds at most[16]. This equilibrium size depends on both the dry volume of any non-volatile material in the DN, and the amount of water retained by that material after evaporative equilibrium is established[17]. The amount of water vapour retained, in turn depends on both the ambient relative humidity (RH) and the hygroscopic salt content of the DN[17–20].

The mechanisms responsible for degrading pathogen infectivity during desiccation are not fully understood but they appear to result partly from increased exposure to chemical and physical agents such as oxygen free radicals and ultraviolet light[21–25]. This increased exposure begins when the respiratory fluid is divided into small droplets during aerosolization, and accelerates when the droplet volume decreases during evaporation.

One important mechanism of chemical degradation is the damage caused by oxidative stress and the generation of oxygen free radicals[26]. The rate at which this damage occurs depends on the defensive capabilities of the microbe and on the composition and size of the residual solute and biofilm remaining in the dried respiratory fluid which surrounds the cell after the initial evaporation phase[21–23, 27, 28]. This residue may act as a protective barrier against cellular desiccation, inhibiting the migration of reactive species, which can damage the cell. The rates at which these processes occur may vary with external (e.g. ambient RH and temperature), and internal factors (e.g. nutrient availability and the rate of microbial metabolism)[25, 28]. The range of timeframes needed to fully explore the infectivity of a bioaerosol may therefore extend well beyond the evaporation time.

The aim of this study was to develop and demonstrate an alternative *in vivo* strategy able to access all timeframes using naturally produced respiratory aerosols to answer questions about the potential for an airborne transmission route for existing and emerging human pathogens. Through this we also sought to contribute to the scant literature on *in vivo* respiratory aging, sampling and culture methods in the context of biological decay.

## Materials and Methods

We have previously described a wind tunnel based “time of flight” technique called the Expired Droplet Investigation System (EDIS)[16] to examine the physical behaviour of respiratory aerosols over very short time intervals (<60 s) following their release[7, 8, 16], however the time of flight approach becomes impractical for durations exceeding 60 s. The need to examine longer aging periods required the adaptation of rotating aerosol storage reservoir techniques pioneered by Goldberg et al. in 1958[29, 30] and further developed by others[31–34] to permit respiratory aerosol to be introduced directly into a rotating aerosol aging chamber.

These time of flight and explicitly timed aging approaches were combined as the Tandem Aged Respiratory Droplet Investigation System (TARDIS), to enable the fixing of aerosol age over very short to very long time frames. The TARDIS therefore consists of two subsystems, a time of flight system known as the TARDIS-Tunnel and an explicitly timed rotating aging reservoir known as the TARDIS-Rotator.

### Experimental Setup

#### TARDIS-tunnel and Rotator systems

The TARDIS-Tunnel, used to study the short-term (t <60 s) aging of the respiratory aerosol is a low velocity wind tunnel that envelops the human subject in a HEPA-filtered particle-free airflow. The airflow transports respiratory emissions from the subject at a well-defined velocity. The aged respiratory emissions are then collected and characterized in the downwind instrument module. This module can be positioned at different sampling locations along the tunnel to vary the aerosol time-of-flight from the subject to the sampling point. A schematic diagram of the TARDIS-Tunnel is provided in Fig B of the S1 File.

The TARDIS-tunnel extends the range previously achievable with the EDIS[16], improves the sample collection efficiency via an isokinetic cone cluster that can be varied to match the desired tunnel air velocity and allows characterization of the emissions with a broader array of instrumentation. The technical details and parameters of the instrument module are described in detail in Section A.1 of the S1 File.

The TARDIS-Rotator is used to study the longer-term aging of the respiratory aerosol (t >60 s). It is a horizontally oriented rotating cylindrical drum equipped with subject and instrument flow management interfaces. The interfaces allow in sequential order: (1) the purging of the drum and the subject’s respiratory tract with HEPA-filtered particle free air; (2) the injection of respiratory aerosol directly from a human subject without contamination by room air, and; (3) the aging and subsequent extraction of that aerosol for characterization.

Technical details concerning the rotating drum, inlet and outlet ports, sensor integration, the drum rotation rate, aerosol handling and the subject and instrument interfaces are given in Section A.2 of the S1 File. A schematic diagram is provided in Fig E of the S1 File.

Test duration limits are also discussed in the S1 File. These limits ensure high breathing air quality, while constraining the sample humidity and temperature, so that aerosol aging occurs under realistic room air conditions. They also restrict the fraction of respiratory aerosol particles re-inhaled by the subject in order to limit associated particle loss from the sample.

HEPA filtered room air was chosen as the background air source in the tunnel and rotator because this purely mechanical filtration technique efficiently removes all particles from the air while preserving the air’s gaseous makeup. Hence, this approach does not interfere with naturally present gaseous oxidative species that may influence the lifetime of viable airborne pathogens. The room air particles removed by filtration could contain particulate phase oxidative species however such material, being bound to the particle, is unable to interact directly with the newly introduced respiratory aerosol particles that contain the pathogen of interest[35, 36].

#### Instrumentation

Both the TARDIS-tunnel and Rotator can accommodate a broad array of instrumentation. The instruments used in the experiments presented here included an Optical Particle Counter (OPC, Lasair II 110, Particle Measuring Systems, Boulder CO, USA), a 6-stage Andersen Viable Cascade Impactor (ACI, Thermo Fisher Scientific, Waltham, MA USA) and dual thermohygrometer probes (HC2-C04, Rotronic Instrument Corp, Bassersdorf Switzerland). The TARDIS-rotator was also fitted with an ultrasonic flow meter (USF, Thor Medical Systems, THRUD medical flow meter), which recorded bidirectional flow at 0.01 s intervals. The USF recorded the flow of HEPA-filtered replacement air into the rotating reservoir as the subject inhaled from the reservoir, and the flow of displaced air out of the rotating reservoir as the subject exhaled or coughed into the reservoir.

The OPC classifies particles according to their laser light scattering behaviour into 6 size channels with lower diameter thresholds at 0.1, 0.2, 0.3, 0.5, 1.0 and 5.0 μm. This instrument has a high sample flow rate of 28.3 L.min^-1^, permitting very low particle concentrations to be measured in real time, which is needed because even under controlled conditions, respiratory aerosols are present at low levels in comparison to ambient aerosols.

The ACI draws 28.3 L.min^-1^ of air and classifies airborne microbe-laden droplet nuclei into six size channels having lower aerodynamic diameter thresholds of 7.0, 4.7, 3.3, 2.1, 1.1 and 0.6 μm[37]. Each of these six size fractions is impacted onto one of 6 agar plates loaded with a growth medium selective for the organism of interest. Specific media were selected for use in the ACI to preserve and culture the bacteria of interest[38, 39]. A range of other techniques including liquid impingement devices such as the Coriolis μ (Bertin Technologies)[40] can be adapted to work with the TARDIS.

The thermohygrometers continuously recorded temperature, humidity and water vapour concentration measurements in both the sample and supply (i.e. dilution) air of the TARDIS-tunnel and Rotator. These water vapour concentration measurements were used to determine the amount of supply air, which was mixed with the aerosol exhaled by the subject.

Four additional measurement techniques intended for future use (not used in the current study) were accommodated in and therefore influenced the design in terms of the required aerosol capacity of the TARDIS-rotator. These were the Biosampler (SKC, PA USA) which draws aerosol at 12.5 L.min^-1^, and consumes 63 L of aerosol; the Ultra Violet Aerodynamic Particle Sizer (UV-APS model 3312, TSI, Shoreview MN USA), drawing 5 L.min^-1^ and consuming 25 L[41]; the Scanning Mobility Particle Sizer (SMPS) (Model 3936, TSI, Shoreview MN USA) drawing 1 L.min^-1^ and requiring 10 L of aerosol [8, 42, 43]; and a tandem differential mobility analyser (TDMA) [44–48] drawing aerosol at 2 L.min^-1^ and requiring 60 L of aerosol.

**Fig 1:**
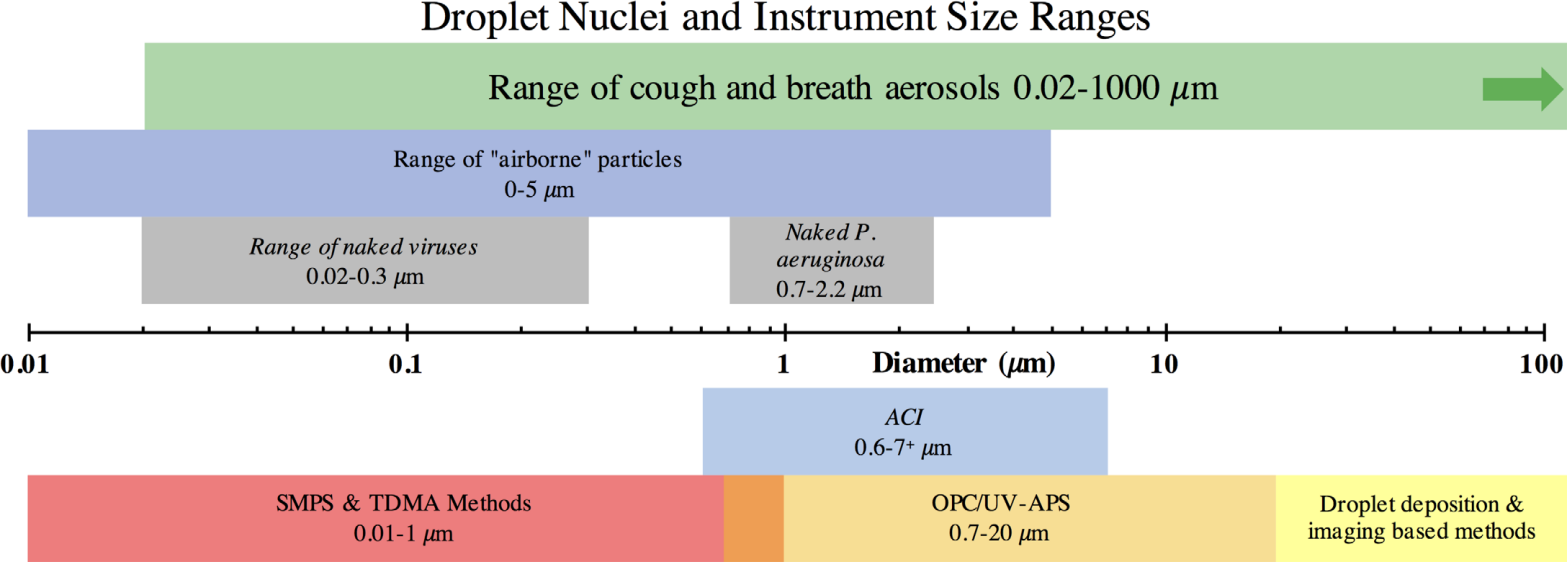
Relationship between respiratory aerosol and instrumentation size ranges and bacterium and virus dimensions. Only the ACI and OPC techniques were employed in the current study.

Fig 1 summarises the particle size ranges of each of the instruments and techniques accommodated by the TARDIS, the sizes of the aerosols present in respiratory emissions and the sizes of bacteria and viruses potentially present in human respiratory aerosol (or more specifically its airborne size fraction).

### Experimental Procedure

During the TARDIS-tunnel tests, the subjects performed a 300 s voluntary coughing and breathing manoeuvre inside the Tunnel’s subject module. Aerosol characterization and collection was simultaneously performed at the instrument module located a set distance downwind of the patient. The measurements presented here were conducted using the OPC and ACI. The distances chosen for the tests were 1, 2 and 4 metres so that for the 0.1 m s^-1^ air velocity setting used in the tunnel, the resulting times of flight (aerosol ages) upon collection were 10, 20 and 40 s respectively. Tunnel blanks were conducted using an identical procedure to the subject tests, but without a subject being present in the tunnel.

For the TARDIS-rotator tests, the rotating chamber was first purged with HEPA-filtered particle free air. The subject then breathed a continuous supply of HEPA-filtered room air for 120 s through a mouthpiece before commencing a 120 s voluntary coughing (i.e. coughing as frequently and intensively as comfortably sustainable) manoeuvre. The manoeuvre was performed through the mouthpiece, via which air was inhaled from and exhaled into the rotating chamber. The rotating chamber was then isolated, and the aerosol inside was allowed to age before undergoing analysis by OPC, and thermohygrometer and size classified collection onto growth media by ACI. As with the TARDIS-tunnel, rotator blanks were conducted using an identical procedure to the subject tests but without a subject present. For a detailed description of the procedures see Sections B and C of the S1 File.

Quantitative Andersen Viable Cascade Impactor bioaerosol cultures were undertaken using chocolate bacitracin (300 mg.ml^-1^) agar incubated aerobically at 35°C for 72 hours [38]. Quantitative and qualitative sputum cultures were performed using horse blood, chocolate bacitracin (300 mg.ml^-1^), MacConkey Agar No. 2, mannitol salt and *Burkholderia* cepacia selective agars incubated aerobically (or 5% CO_2_ where appropriate) at 35°C agar for 72 hours. A representative selection of presumptive *Pseudomonas aeruginosa* colonial morphotypes were selected from the primary cultures, screened for cytochrome c oxidase and growth at 42°C, and then confirmed as *P*. *aeruginosa* using duplex real-time polymerase chain reaction PCR[49]. Confirmed bioaerosol and sputum isolates were thereafter genotyped using enterobacterial repetitive intergenic consensus-PCR (ERIC-PCR) and cluster analysed alongside a previously characterized panel of Australian shared *P*. *aeruginosa* strains[50].

### Subjects

The challenge aerosol used to test the TARDIS was produced by two male patients with cystic fibrosis (CF); (aged 23 and 27 years) and chronically infected with *P*. *aeruginosa*. Both were members of a larger cough aerosol study cohort[39]. Subjects were recruited from the adult CF Centre at The Prince Charles Hospital (TPCH), Brisbane, Australia. Exclusion criteria included recent pneumothorax and haemoptysis. Subjects S1 and S2 were randomly selected for additional analysis, from the ten highest producing patients in larger cough aerosol study cohort[39]. Patients vary widely in their production of viable airborne *P*. *aeruginosa* bearing DN and in some cases this limits the level of analysis which can be performed. For example, for low producing patients it is not possible to clearly resolve and characterize dual mechanism, pathogen decay behaviour. The selection of patients with high cough aerosol production permitted a clear demonstration of the more detailed analysis, which can be achieved using the TARDIS with high viable DN production rates.

Both subjects attended two non-successive (to allow for recovery) study days, one for the TARDIS-tunnel test and one for the Rotator test. A sputum sample was used to confirm infection status at the time of testing. These two subjects, designated S1 and S2 in the current analysis, corresponded to subjects S15 and S7 of our previous publication[39]. S1 was a 23 year old male and had moderately-severe bronchiectasis (FEV_1_−57.7% predicted), undernutrition (BMI 20.9 kg.m^-2^), and chronic *P*. *aeruginosa* infection. This subject had a rapid and sustained clinical decline over the 5 years prior to study and had completed an intravenous antibiotic treatment course (IV Meropenem 1 g, tds and IV Tobramycin 320 mg od) for a pulmonary exacerbation 6 days prior to his first study. Subject S1 had mucoid and non-mucoid *P*. *aeruginosa* cultured in the sputum on the day of the study and in the prior 12 months. In addition, *Stenotrophomonas maltophilia* was isolated on one occasion 9 months prior to the study date. S2 had moderately-severe bronchiectasis (FEV_1_−50.9% predicted), undernutrition (BMI 24.8 kg.m^-2^), and chronic *P*. *aeruginosa* infection. This subject was clinically stable over the 5 years prior to and at the time of study (17/912) with the most recent course of intravenous antibiotics administered 5 weeks prior to the study date. Subject S2 also had evidence of mucoid and non-mucoid *P*. *aeruginosa* cultured in the sputum on the day of the study and only mucoid *P*. *aeruginosa* in the prior 12 months.

The study was approved by the Queensland Royal Children’s Hospital Human Research and Ethics Committee (HREC/11/QRCH/44), The University of Queensland Medical Research Ethics Committee (2012000615) and the Queensland University of Technology University Human Research Ethics Committee (1100000618). Written consent was obtained from both subjects. Cough manoeuvres were supervised by a healthcare professional and emergency equipment was available in the event of an adverse event.

### Data Analysis

The concentration of viable bacteria in the aerosol was based on the number of colonies appearing on the ACI agar plates during incubation. Total colony forming unit counts (CFU) on each agar plate of the ACU were obtained by counting the number colonies present and then applying a statistical coincidence correction for overlapping colonies[37, 51]. The corrected CFU count was then divided by the volume of air sampled by the ACI to obtain the concentration of viable bacteria in the aerosol.

#### Dilution factor (DF) normalization

Dilution factor (DF) normalization of the measured total and viable aerosol concentrations was used to estimate the undiluted concentrations in the breath. This normalization consisted of multiplying the concentrations measured in the sample by a breath dilution factor. For viable and total particles these undiluted concentrations represented the concentrations that would have been obtained if the undiluted breath had been aged at the humidity and temperature of the diluted sample. DF normalization effectively eliminates aerosol concentration variance caused by differences in dilution between individual tests within each, and across both, of the two aging systems. The DF normalized concentrations can therefore be directly compared to reveal differences in total and viable aerosol production in experiments involving different subjects, and/or using different aging intervals.

Two different methods were used to determine DFs for the samples. Firstly, a water vapour based method derived the DF from the dilution of the vapour present in the exhaled breath. Secondly, a flow-based method was used to obtain an estimate of DF from measurements of the volume flows of exhaled breath and room air which were combined (at constant temperature and pressure) to create the sample. For practical reasons the latter method could not be implemented in the TARDIS-tunnel system, but both methods were applied in the Rotator to provide validation of the humidity-based approach. Comparison of the water vapour and flow-based measurements of dilution showed that the water vapour based technique was accurate to within ±15%. The detailed rationale and approach taken to correct the TARDIS-tunnel and Rotator measurements for dilution are described in Section E of the S1 File. Examples of the dilution factor measurement and the validation of the water vapour based method are also presented there.

#### Total aerosol (TA) normalization

Where analysis focussed specifically on the behaviour of the bacteria, total aerosol (TA) normalization was preferred over DF normalization because it produced a direct measure of the aerosol’s bacterial viability independent of the subject’s aerosol production rate. Like DF normalization, the TA normalization approach removed dilution-associated variance but it offered the added advantage of removing variance associated with aerosol production and deposition loss. TA normalization consisted of dividing the CFU concentrations by the corresponding total aerosol concentrations to obtain the number of CFU per particle. In an extension of this approach, CFU concentrations could also be divided by the total aerosol volume concentration (calculated using the aerosol size distribution measured by the OPC) to obtain the number of CFU yield per unit volume of sputum.

#### Alignment of the OPC and ACI data

The calculation of the size distribution of CFU yield per droplet nucleus required that the OPC and ACI data were aligned on a size basis and presented using a common scaling. To achieve alignment of the OPC data size ranges with those of the ACI, the OPC dCn versus OPC lower channel boundary (D_L-OPC_) data was rescaled as dCn/dLogD at each of the OPC channel geometric mean diameters (D_GMD-OPC_). A curve fit to the dCn/dLogD data could then be remapped to the ACI channel GMDs (D_GMD-Imp_) via interpolation. The resulting ACI channel based size distribution was then rescaled as dCn versus the corresponding ACI channel lower boundaries (D_L-Imp_) to determine the concentrations which impacted each ACI plate.

## Results

Results concerning the TARDIS’s performance and the aging of pathogen-laden aerosols produced by two individuals with CF (S1 and S2), and chronically infected with *P*. *aeruginosa*, are presented below. The cough aerosols were aged for 10, 20, 40, 300, 900 and 2700 s as follows. For short term aging, two repeated tests denoted test A and test B were conducted in the TARDIS-tunnel at distances of 1m, 2m and 4m, which corresponded to aerosol aging intervals of 10 s, 20 s and 40 s respectively. For long-term aging, two repeated tests were conducted in the Rotator at aerosol ages of 300 s and 900 s, and a single test was conducted at 2700 s of aging.

### Breath dilution

Table 1 shows the sample dilution conditions that occurred in the TARDIS-tunnel and Rotator. The resulting DF ranged from 9 to 15 in the TARDIS-rotator and from 22 to 46 in the TARDIS-tunnel. The DF was considerably more variable in the tunnel and showed a gradual increase with distance from the subject due to the increasing dispersion of the cough plume.

**Table 1:** Dilution factors for each TARDIS-Tunnel and TARDIS-Rotator experiment.

Test Type →	TARDIS-Tunnel						TARDIS-Rotator					
Age (s)	0.1		0.2		0.4		300		900		2700	
Distance (m)	1		2		4		NA		NA		NA	
Sample	A	B	A	B	A	B	A	B	A	B	A	NA
Subject S1	36	30	41	33	46	36	13	12	9	15	14	NA
Ave/SD	33/3.7		37/5.5		41/6.9		12/0.8		12/4.5		14/(NA)	
Subject S2	22	23	29	26	29	28	10	12	9	9	9	NA
Ave/SD	22/0.33		27/2.0		29/0.84		11/1.5		9/0.01		9/(NA)	

Dilution also differed between the two subjects, being consistently higher for S1 than S2 in both the tunnel (S1 being 42% higher than S2) and the rotator (S1 being 31% higher). This was caused by marked differences in the two subjects’ mean ventilation rates during the coughing and breathing manoeuvre, with S1 coughing and breathing at mean flow rates of 14.8–17.4 L.min^-1^ as measured by the ultrasonic flow meter during rotator tests, compared to S2 coughing and breathing at 19.0–23.5 L.min^-1^. This led to a smaller contribution of the breath being mixed into the samples obtained from S1 than those from S2, and hence a higher dilution factor for S1. Dilution was found to be more consistent across multiple tests for the rotator because the exhaled breath was always more fully captured within that apparatus.

### Aerosol size distributions

**Fig 2:**
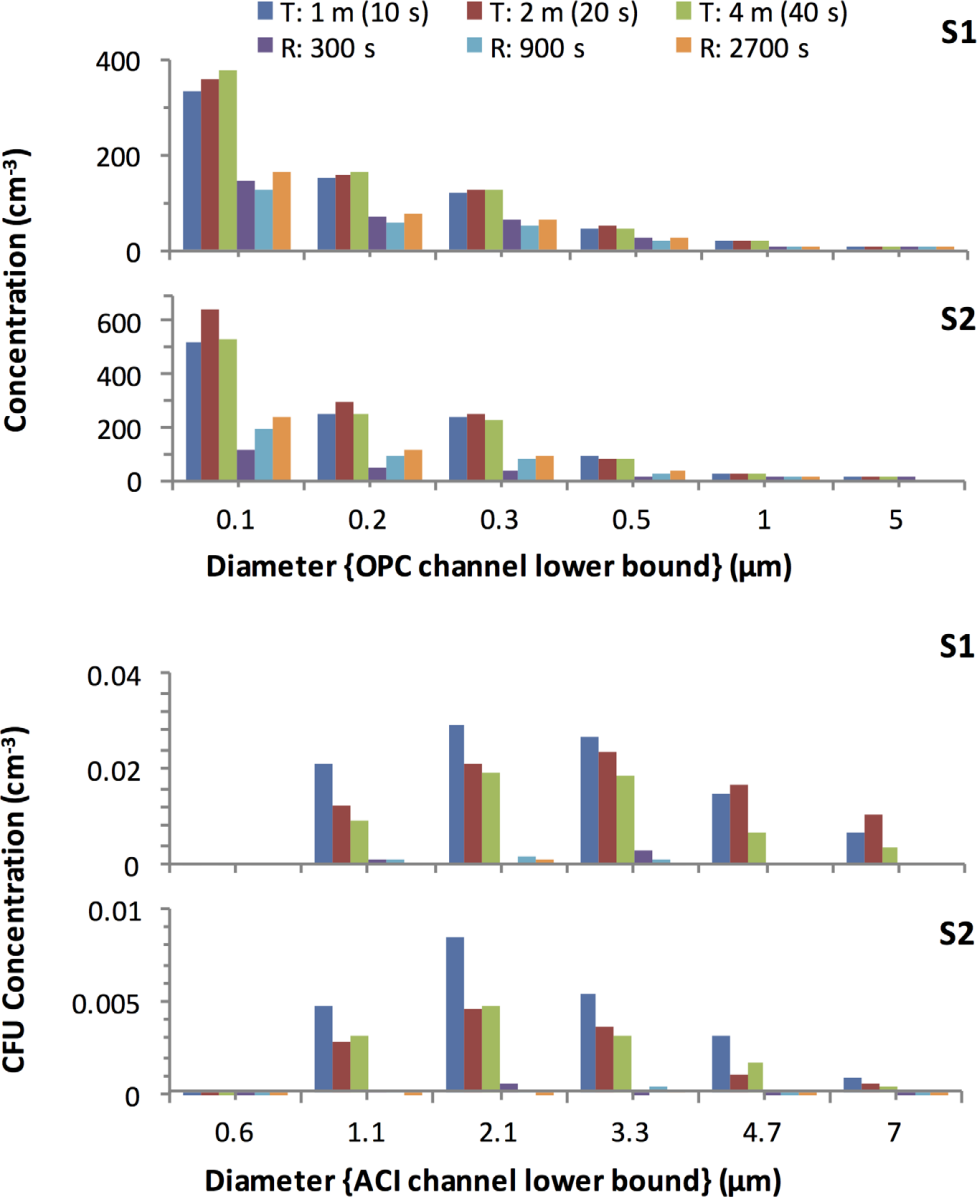
TARDIS-tunnel and Rotator based measurements of the size distribution of the aerosol for subjects S1 and S2. The concentrations are DF normalized to the point of origin in the respiratory tract and represent average concentrations within each OPC and ACI size channel. They are based on measurements recorded by the OPC and ACI. Diameters represent the lower boundaries of each OPC and ACI size channel. Measurements in the tunnel were at distances of 1 m (10 s of aging), 2 m (20 s) and 4 m (40 s). Those in the rotator were made after durations of 300 s, 900 s, and 2700 s. Note that the horizontal axis scale differs in the upper and lower panels.

Fig 2 shows the average dilution corrected size distributions recorded by the OPC and ACI over two successive experiments using the TARDIS-tunnel and the TARDIS-rotator. The uncorrected data are presented in Figs M and N of the S1 File.

#### Aerosol production variance in the TARDIS

**Fig 3:**
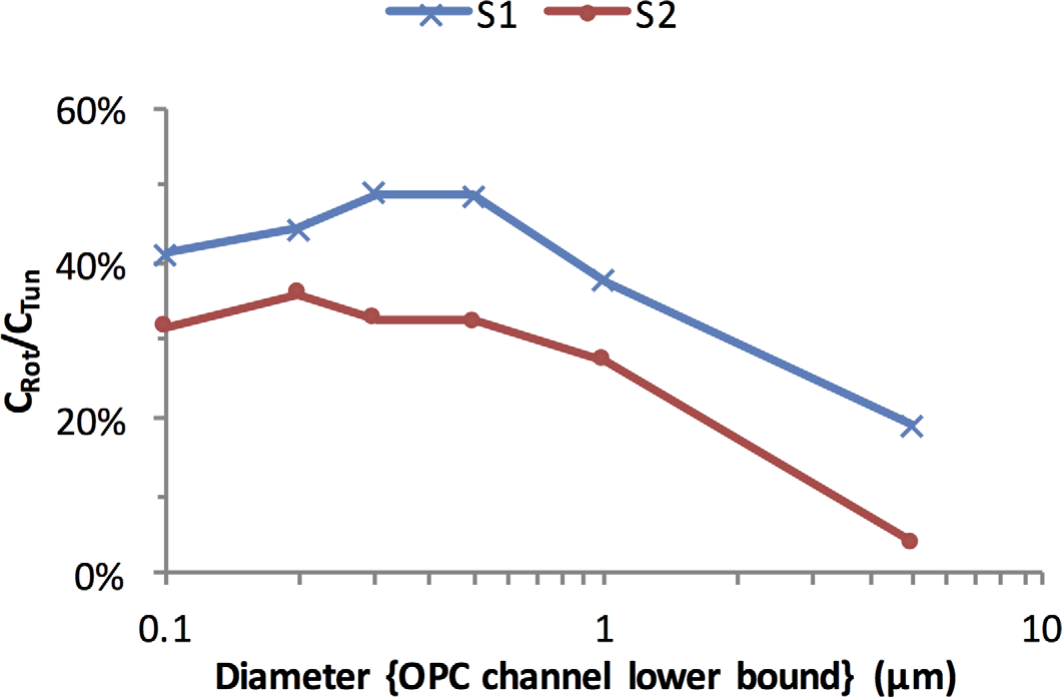
Ratio of the mean of the normalised aerosol concentrations produced when using the TARDIS-rotator to that when using the TARDIS-tunnel.

Differing levels of dilution between the two systems were not the sole source of difference in concentration between TARDIS-tunnel and Rotator tests as revealed by the DF normalized concentrations, (Fig 2). The effect of dilution was removed by normalization, yet the normalized concentrations still showed consistent differences between TARDIS-tunnel and Rotator measurements for both subjects. Fig 3 shows that the production efficiency when coughing in the TARDIS-rotator was consistently only 40% of that in the Tunnel for diameters up to 1 μm, and it fell to 10% at the largest measured sizes. It is possible that some moderation of the cough strength occurred when the subject used a mouthpiece and nose clip to cough into the drum when compared with unencumbered coughing in the Tunnel.

#### Total aerosol normalisation to eliminate dilution and production variance

**Fig 4:**
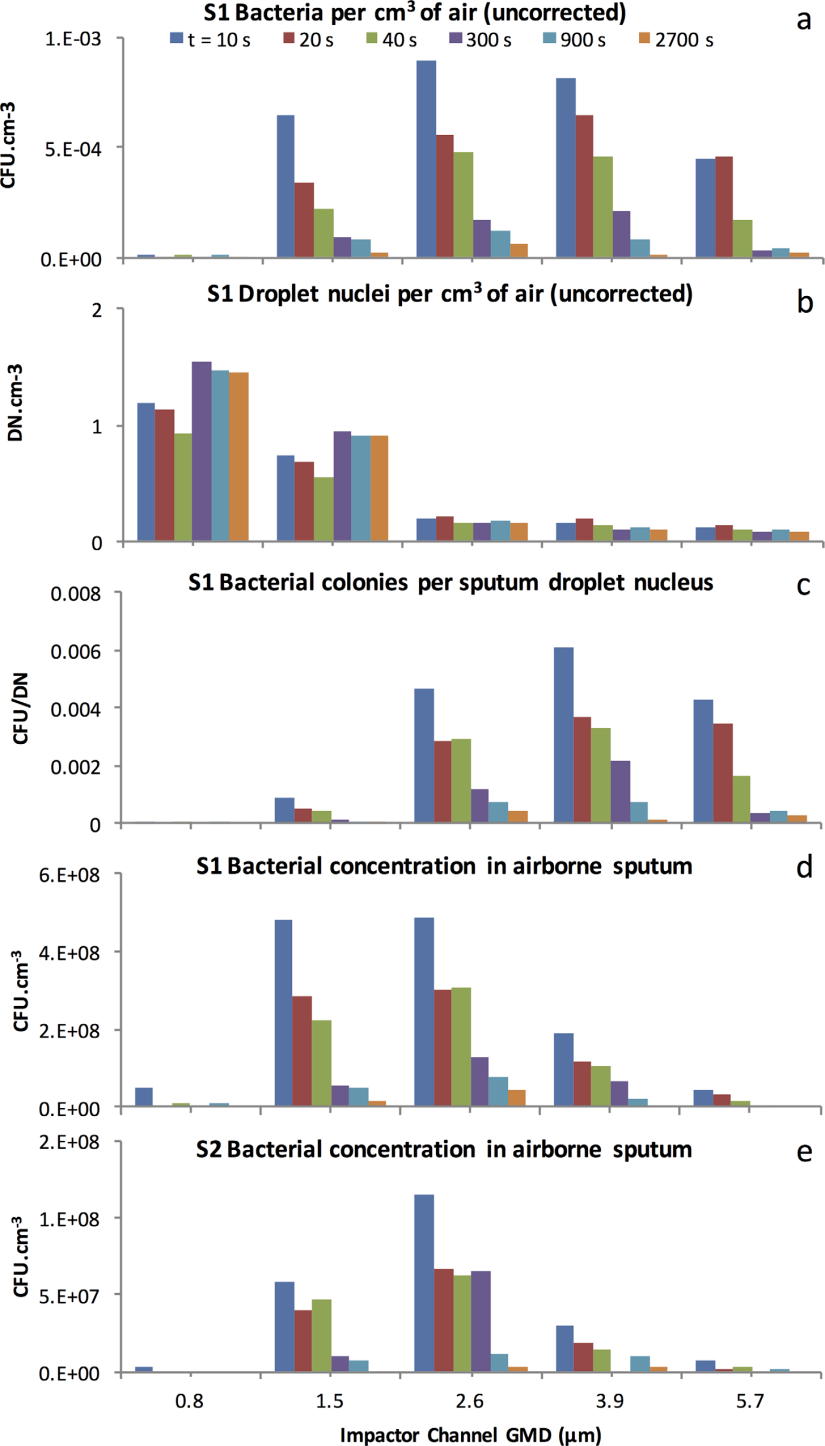
(a) Viable (colony forming) DN counts *per cm*^*3*^ *of sample air* versus DN size for subject S1. (b) Total DN count *per cm*^*3*^ *of sample air* versus DN size for S1. (c) TA normalized viable DN counts (expressed as CFU *per DN*) for S1. (d and e) TA normalized viable DN counts (expressed as CFU *per cm*^*3*^ *of airborne sputum*) for S1 and S2. Each colour represents a different aging interval. Note that the vertical axis scale varies according to the variable represented.

The viable *P*. *aeruginosa* laden DN number concentrations in each ACI size class in the diluted cough aerosol after aging for 10, 20, 40, 300, 900 and 2700 s are presented for subject S1 in Fig 4A. Corresponding total DN number concentrations are shown in Fig 4B. The TA normalized values are effectively the ratio of these previous two size distributions expressed as the number of CFU yielded per DN as shown in Fig 4C. Fig 4D shows the same TA normalised values, after scaling by the particle volume. This represents the concentration of viable *P*. *aeruginosa* in the sputum at each DN size or CFU yield per unit airborne sputum volume. Fig 4E shows the equivalent result for subject S2.

## Discussion

### Performance of the TARDIS

After developing and testing the system, we can report that: (1) The TARDIS provided a practical *in-vivo* approach to aging respiratory aerosols that permitted both short and long-term aging. The aerosol was collected directly from a human subject into particle free air and aged at room air temperature and humidity for 10–2700 s (or longer). (2) Contamination from other (e.g. non-respiratory) aerosol sources was carefully excluded throughout collection, aging and sample characterization, and verified by numerous blank sample characterizations. Cultures from samples collected without a subject present showed no evidence of colony formation. Similarly we were able to use the OPC prior to and after sample extractions to show that no room air contamination occurred (Sections B and C of the S1 File). (3) Dilution of the exhalation was calculated from the dilution of the exhaled water vapour to an accuracy of ±15%, as verified by an independent measurement technique based on exhaled air and dilution air contributions to the sample. (4) The system accommodated a full suite of droplet nuclei characterization instrumentation, chosen to provide comprehensive size and concentration measurement across the airborne size range, and beyond. More broadly, our system represents a useful means to accurately probe the physical and biological characteristics of human respiratory aerosols, that has applications in addressing the relatively poorly defined role of such aerosols in communicable infection.

Patient safety was carefully maintained through appropriate measures built into the design and protocols. These measures ensured the maintenance of appropriate CO_2_ and O_2_ levels within the system, limited the duration of subject testing, eliminated potential sources of cross infection and ensured suitable patient supervision by trained health professionals. Exclusion of contamination during sample collection, characterization, extraction, and handling was carefully verified through the previously described blank sample characterization procedures. No *P*. *aeruginosa* or other microbes identified in cough aerosols were isolated from the surface swabs or blank aerosol samples. However numerous colonies were found in identical treatment of cough aerosol from infected patients. The *P*. *aeruginosa* genotypes isolated from the cough aerosols of each patient were also 100% concordant with those cultured from the corresponding sputum samples[39].

### CF patient versus healthy subject cough aerosol concentrations

While insufficient to draw definite conclusions for the patient cohort as a whole given the small sample size, our findings for these two patients strongly suggest that measurements of cough aerosol production obtained from healthy subjects may be an inadequate basis for predicting airborne infection transmission risk. Individuals with CF were found to produce far greater quantities of aerosol when they cough than healthy subjects previously tested in the EDIS using the UV-APS during coughing[8, 16].

Table 2 shows that the DF normalized (ie undiluted breath) cough aerosol number concentrations for particles of 1μm in diameter and larger were two orders of magnitude greater than the mean DF normalized concentration in the same range as previously recorded using similar techniques for healthy subjects during coughing[8]. The mean concentrations in this size range for subjects S1 and S2 were 20.1 cm^-3^ and 22.5 cm^-3^ for S1 and S2 respectively; while the mean overall for a healthy subject group tested previously was 0.22 cm^-3^[8].

**Table 2:** Mean DF Normalised (undiluted breath) cough aerosol number concentrations for particles of 1 μm in diameter and larger in subjects S1, S2 and a previously studied group of 15 healthy volunteers [8, 16].

	S1	S2	Healthy Controls[8]
Mean	20 cm^-3^	23 cm^-3^	0.22 cm^-3^
SD	2 cm^-3^	3 cm^-3^	0.15* cm^-3^

*Standard deviation across 15 volunteers.

### DN size producing the largest numbers of bacterial colonies (2.6 μm)

DN of different sizes had varying probabilities of delivering viable bacteria. This was evident through a marked difference between the viable (colony forming DN) cough aerosol size distribution (Fig 4A) and total (irrespective of colony formation) aerosol size distribution (Fig 4B) shape and concentration.

The viable DN size distribution, in combination with particle size dependent deposition probabilities in the various regions of the respiratory tract, together determine the likelihood of delivering viable bacteria to a susceptible region of the respiratory tract. In these two subjects the viable DN concentration peaked at a Geometric Mean Diameter (GMD) between 2.6 μm and 3.9 μm. These DN sizes produced the largest number of colonies on the ACI plates as shown in Fig 4A. This outcome resulted from the distribution of total DN concentration versus size as displayed in Fig 4B being modified by the probability distribution of individual DN of each size carrying a viable bacterial load as displayed in Fig 4C. Most of the DN were 1.5 μm and smaller in diameter, but the *P*. *aeruginosa* colonies were produced mainly by DN which were 1.5 μm or larger in diameter.

The probability of deposition in the respiratory tract for particles larger than 1 μm, strongly favours deposition in the uppermost regions of the respiratory tract[52]. Nevertheless, 10–15% of particles in the 1–4 μm range deposit in the alveoli, implying that significant fractions of viable bacteria are likely to be deposited in this region for bystanders inhaling the aerosol[52].

### DN size most likely to contain viable bacteria (3.9 μm)

Smaller DN were far less likely to carry viable bacteria. The size distribution of the *P*. *aeruginosa* CFU yield per DN (Fig 4C) revealed the relative capacity of droplets of different sizes to deliver culturable bacteria. The optimum size for delivery corresponded to the peak of this size distribution and this peak was shifted toward larger diameters relative to the viable aerosol size distribution (Fig 4A). If we were to assume that a single source of aerosol produced all DN from a common fluid reservoir containing a uniform concentration of bacteria, then the DN size most likely to contain a viable bacterium should simply be the largest ones, because these contain the most sputum. This single aerosol source assumption is tested and disproved in section 4.6 below.

### DN size containing the largest concentrations of bacteria (1.5–2.6 μm)

For minimally aged aerosols, the CFU yield per unit volume of airborne sputum (Fig 4D and Fig 4E) was greatest for 1.5 and 2.6 μm droplet nuclei and decreased strongly as the size increased beyond this limit. The decrease in yield at the largest sizes, strongly suggests a secondary source of larger DN in the respiratory tract, which was drawing on fluid that had a lower bacterial load.

The CFU concentration also declined at the small end of the scale, falling suddenly to zero below 1.5 μm (the exact cut off was between 0.8 and 1.5 μm). This cut-off reflects the physical limitation imposed by the size of the *P*. *aeruginosa* organism, which is approximately cylindrical in shape, with a characteristic (geometric mean) diameter between 0.87 and 1.5 μm[53]. This range therefore represents the smallest possible DN containing the bacterium. No such limitation applies to non-viable droplet nuclei.

### Evidence of multiple source regions with different bacterial loads

Consistent with the observation of multiple source regions reported previously for cough aerosol from healthy subjects[7, 8], multiple aerosol source regions with different bacterial loads appear to exist within the respiratory tract, giving rise to different but overlapping droplet size distributions. The evidence for this finding is described in the following three paragraphs.

If a single reservoir of homogenous respiratory fluid had produced DN of all sizes, then larger DN should have contained more bacteria than smaller DN and therefore should have exhibited a correspondingly higher probability of colony formation per DN. Although such a trend began in Fig 4C for diameters up to 3.9 μm, the curve representing CFU/DN versus diameter did not sustain the expected trend beyond that size. This finding implies one of two possibilities: (1) large droplets were inhospitable to the *P*. *aeruginosa* organism; or (2) the single reservoir hypothesis is wrong and at least two distinct fluid reservoirs with different bacterial loads gave rise to two overlapping droplet size ranges (ie two size distribution modes with distinct characteristic diameters).

Possibility 1 is unlikely because the shear forces needed to break a fluid into droplets increase as the droplet size decreases. These shear forces also affect bacteria, so *P*. *aeruginosa* in the smaller droplets are more likely to be damaged during aerosolization. Furthermore, the process of pathogen degradation (at least for gram negative bacteria) while airborne in exhaled aerosol droplets, results partly from oxygen toxicity whereby oxidation by O_2_ and O_3_ can produce toxic products that damage bacterial cells[21–24]. Larger droplets take much longer to evaporate and present a greater barrier to the entry by diffusion, of destructive oxidative chemical species thereby impeding their approach to the embedded bacteria from the surrounding air.

The evidence therefore supports possibility 2, where multiple aerosol source regions with different bacterial loads exist within the respiratory tract such that these aerosol sources give rise to different but overlapping droplet size distributions. This finding is consistent with the observation of multiple source regions reported previously for cough aerosol from healthy subjects[7, 8], because respiratory infection is unlikely to be uniformly distributed throughout the respiratory tract.

### Bacteria were associated with right tail of a broad submicrometer DN size distribution peak extending to diameters larger than 3.9 μm

The increasing concentrations of DN seen at smaller sizes in Fig 4B imply that the production mode for the small DN is quite broad, and is centred in the submicrometer range however most of the resulting DN are smaller than the *P*. *aeruginosa* bacterium.

A complex aerosol generation system such as the human respiratory tract during coughing is likely to include multiple sources of aerosol and each of these will have its own characteristic aerosol size distribution mode[7, 8]. These modes combine to produce a multimodal aerosol size distribution, consisting of overlapping lognormal curves (one for each production mode), each with a characteristic median diameter and spread.

Again comparing Fig 4D and 4E to Fig 4B, it can be concluded that the fluid feeding the production of the smallest DN must have been far richer in bacteria than that which fed the distinct process that produced most of the larger DN. This second production mechanism is not resolved in the size distribution of Fig 4B, due to a lack of sufficient size resolution in the OPC measurements. Nonetheless it appears to have produced significant numbers of bacteria-poor DN in the 3.9 μm to 5.7 μm range in order to have reduced the mean concentration of bacteria in the airborne sputum for DN of those sizes to well below the concentration seen at the smaller diameters, where the submicrometer mode dominates the population of DN. The increasing concentrations of DN seen at smaller sizes in Fig 4B imply that the production mode for the small DN is quite broad, and is centred in the submicrometer range.

### Bacteria were associated with aerosol from the lower respiratory tract

It is therefore probable that at least two aerosol-generation processes were occurring at different regions of the respiratory tract. We have previously shown that multiple droplet production mechanisms produced progressively larger characteristic droplet sizes at higher points in the healthy respiratory tract[8]. The observation, which is reported for the first time, of a decreasing CFU yield per unit volume of respiratory tract fluid with increasing DN diameter, suggests (assuming that the sites of aerosol production are similar to those in healthy subjects) that the viable concentration was smaller in the middle and upper regions of the respiratory tract of these patients. Active bacterial shedding therefore appears to occur predominantly in the lower respiratory tract in at least these two patients. Using an *in vitro* system Clifton and colleagues demonstrated that *P*. *aeruginosa* strains expressing a mucoid phenotype showed a survival advantage within aerosols when compared to non-mucoid strains[54]. In the current study we isolated mucoid and non-mucoid morphotypes from the aerosol cultures in both subjects. However, given the small sample size, the nature of the model, the presence of mixed *P*. *aeruginosa* morphotype infections, and a predominance of the mucoid phenotype in both sputum and aerosol cultures we were unable to determine if there were any differences in survival associated with colonial phenotype or biofilm mode of growth.

### Two decay processes degrade bacterial viability

The decay of bacterial viability was very rapid in the first seconds of the aerosol’s release but then slowed dramatically as the droplets aged. This result is comparable to a dual decay process previously reported for aerosolised *E*. *coli*[23].

**Fig 5:**
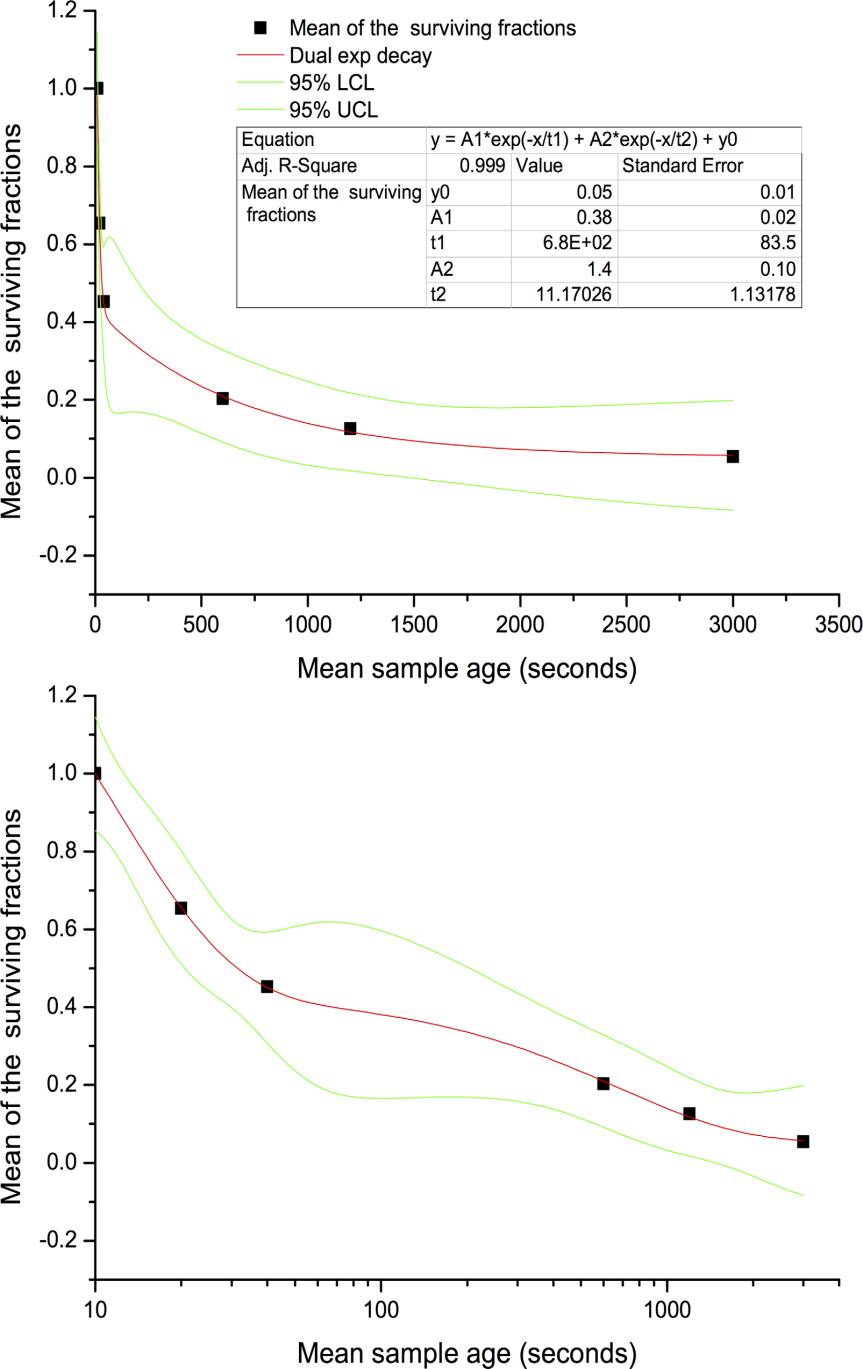
Dual decay curve fit for subject S1 shown using a linear (graph at top) and log10 time axis (bottom). Vertical axis shows the mean fraction of the CFU volume concentration relative to that at age 10 s. “Mean of surviving fractions” is the mean across all droplet sizes for subject S1. Mean sample age is the mean DN age during ACI extraction.

The orderly biological decay process occurring in the aging DN can be seen in Fig 5. Fitting an exponential model revealed a dual exponential decay processes involving two distinct half-lives; one of less than 10 s and a second of the order of 10 min. Similar dual decay processes have been observed previously. Benbough[23], found that aerosolized *Escherichia coli* showed an initial very rapid death rate, followed by a much slower secondary one. The initial rapid decay was very sensitive to the RH and oxygen content of the atmosphere in the aging reservoir while the slower decay was not. Replacing air in the aging reservoir with nitrogen almost eliminated the rapid death process highlighting the role of oxygen toxicity.

Benbough’s *E*. *coli* experiments were conducted using a time resolution of 2.5 minutes or larger, so the decay rate for the faster process was not resolved. The initial rapid decay for *E*. *coli* exhausted itself between aerosol injection and the first 2.5 minute sample extraction making a half life of the order of seconds plausible (the author described this decay as instantaneous). The secondary decay process had a half-life also of the order of 10 minutes. The time frames over which these decay processes played out appear to be comparable to those observed in the current study. In any case the overlapping environmental mechanisms responsible for the biological decay of *E*. *coli* might also apply to *P*. *aeruginosa*.

We wish to emphasise that these results (S2 also displayed dual decay) represent only two high producing individuals selected from the full patient sample group. They were chosen to facilitate a clear illustration of the utility of the aerosol collection methods used in our study. The results therefore should not be taken as being representative of the wider patient group, which as shown in our previous publication, displayed an overall mean bioaerosol biological decay half-life of 50 minutes[39].

### Slow decay is most relevant to potential airborne infection risk

It should also be noted that the very rapid initial decay process demonstrated here is relevant to the study of mechanisms of biological decay in aerosol during the very early stages of aerosol formation where dispersion is dominated by the explosive release from the respiratory tract. Such rapid decay is perhaps of more limited interest when modelling the control of airborne infection risk in the room air environment because the dispersion and air exchange processes that influence exposure tend to act over longer time frames of the order of minutes not seconds. It is therefore primarily the long-term decay (the focus of our previous paper[39]) that is likely to influence any potential airborne transmission risk.

**Fig 6:**
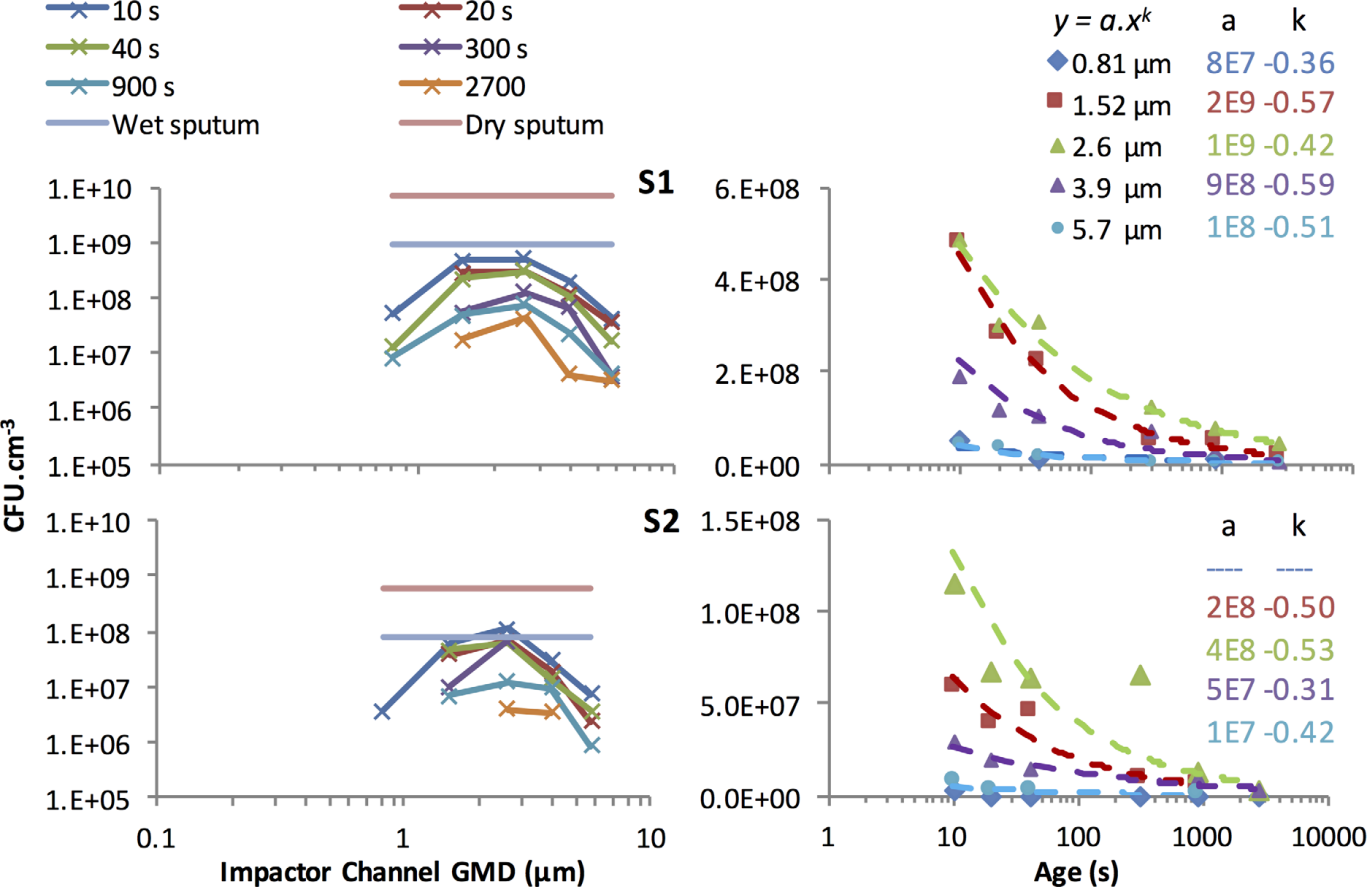
Left: The decay of the sputum aerosol CFU yield per unit of nominal sputum droplet nucleus volume versus time for S1 (top left) and S2 (bottom left). Right: The CFU yield per unit volume of aerosol sputum material for S1 (top right) and S2 (bottom right). Also shown (left, flat lines) are the measured CFU yield per unit volume of wet sputum from each subject and the estimated CFU yield for that sputum if dried to the same degree as the aerosolised sputum. Also shown (right dashed curves) are power law curves fitting the behaviour.

The size distribution of the CFU yield per unit DN volume for the two subjects is shown in the two graphs to the left of Fig 6. Both subjects produced a clear maximum yield per unit volume of sputum near 2.5 μm. The concentration at this peak was similar to the measured CFU yield per unit volume of conventionally collected sputum obtained from the patients. Allowing for the fact that the DN, unlike the sputum sample, had dried and therefore shrunk in volume while airborne, the maximum CFU yield per volume in the aerosol after 10 s of droplet aging was of the order of 10% of that in the sputum sample. This implies that 90% of the organisms may have lost viability within 10 s, which is consistent with the observed decay rate revealed at the right hand side of the figure showing the decay behaviour at each DN size. Note that the size specific mean concentration data points displayed greater variance than was the case for the means taken across all DN sizes as used to examine the dual decay profile in Fig 5. The decay profiles in Fig 6 were therefore fitted with simple power law functions rather than dual exponential functions.

### Summary of findings

The study produced a number of significant findings concerning cough aerosols produced by individuals with CF and chronically infected with *P*. *aeruginosa*. Some of these findings are summarised in Table 3.

**Table 3:** Summary of findings concerning *P*. *aeruginosa* laden cough aerosol from the two subjects.

Aerosol size distribution peaks		Bacterial content of aerosol DN		Bacterial half life	
Total DN	Viable DN	Largest in-sputum bacterial concentrations	DN size most likely to contain bacteria	Short	Long
100 nm	2.6 μm	1.5–2.6 μm	3.9 μm	<10 s	>10 min

The viable (colony producing) cough aerosol concentration peaked at 2.6 μm. The majority of the DN were smaller than 1 μm in diameter, but *P*. *aeruginosa* colonies were produced primarily by 1.5 μm or larger DN.

The aerosol size distribution peak was very broad, and was centred in the submicrometer range, but the smallest and most numerous DN were too small to carry a *P*. *aeruginosa* bacterium. The DN producing the largest numbers of colonies per unit volume of airborne sputum matter, were between 1.5 and 2.6 μm in diameter. These appeared to arise from the leading edge of the broad submicrometer mode.

DN larger than 3.9 μm appeared to contain smaller concentrations of viable bacteria, supporting the existence of multiple aerosol sources within the respiratory tract, producing aerosol from fluid reservoirs containing different viable bacterial concentrations.

Two decay processes with very different half-lives acted to reduce bacterial viability. These half lives were consistent with a dual decay process previously reported for aerosolised *E*. *coli*[23].

## Supporting Information

S1 File Supplement File. (DOCX)
