# Supplementary material for: A Novel Method and Its Application to Measuring Pathogen Decay in Bioaerosols from Patients with Respiratory Disease
Source: PLoS One. 2016 Jul 7;11(7):e0158763. doi: 10.1371/journal.pone.0158763 (PMC4936712; doi:10.1371/journal.pone.0158763)
Supplement: S1 File — (DOCX) [file pone.0158763.s001.docx]

# Supplement File

# A novel method and its application to measuring pathogen decay in bioaerosols from patients with respiratory disease

1. Graham R Johnson^1*^, Luke D Knibbs^2^, Timothy J Kidd^3,4^, Claire E Wainwright^5,6^, Michelle E Wood^7,8^, Kay A Ramsay^5,7^, Scott C Bell^5,7,8^, Lidia Morawska^1^
2. ^1^International Laboratory for Air Quality and Health (ILAQH), Queensland University of Technology (QUT), Brisbane, Queensland, Australia
3. ^2^School of Public Health, The University of Queensland, Herston, Queensland, Australia
4. ^3^Child Health Research Centre, The University of Queensland, Herston, Queensland, Australia
5. ^4^Centre for Experimental Medicine, Queen’s University, Belfast, Northern Ireland, United Kingdom
6. ^5^School of Medicine, The University of Queensland, Herston, Queensland, Australia
7. ^6^Department of Respiratory and Sleep Medicine, Royal Lady Cilento Children’s Hospital, South Brisbane, Queensland, Australia
8. ^7^Lung Bacteria Research Group, QIMR Berghofer Medical Research Institute, Herston, Queensland, Australia
9. ^8^Department of Thoracic Medicine, The Prince Charles Hospital, Chermside, Queensland, Australia
10. ^*^Corresponding Author
11. E-Mail: [g.johnson@qut.edu.au](mailto:g.johnson@qut.edu.au)

# TARDIS

The main elements of the TARDIS appear in the images within Fig A of the S1 File below.

Fig A of the S1 File: TARDIS (clockwise from top left): (a) TARDIS-tunnel and Rotator; (b) Tunnel side view showing subject, Andersen Viable Cascade Impactor (ACI) and Ultra Violet Aerodynamic Particle Sizer (UV-APS); (c) Tunnel interior looking upwind from instrument inlet cone toward the subject; (d) Rotator cabinet interior; (e) Rotator Subject Interface.

## TARDIS-tunnel:

The TARDIS-tunnel shown schematically in Fig B of the S1 File is designed to provide a radially uniform velocity profile so that the age of the aerosol at the sample extraction point is not dependant on the vertical or horizontal changes in the position of the subject’s head in the subject module. A typical velocity profile in the tunnel is given in Fig C of the S1 File, in this case with a velocity setting of 0.4 m.s^-1^. The velocity was found to vary by 12 % over the entire traverse, however the positioning of the subject varies by only a small fraction of this range and an appropriate estimate of the variation within the utilised portion of the emission plane would be less than 5 %. The achievable distances and durations for TARDIS-tunnel are given in Table A of the S1 File.

Fig B of the S1 File: Schematic diagram of the TARDIS-tunnel. *Figure published previously[*[*1*](#_ENREF_1)*], reprinted with the permission of Elseviere.*

Fig C of the S1 File: Mean velocity profile across the TARDIS-tunnel

Table A of the S1 File: The TARDIS-tunnel configuration options. The modular design allowed for a variable mouth to cone distance using modules of various lengths.

| Module Number | | | | | | | Mouth to Cone Distance (mm) | Durations V_air_=100mm/s |
| --- | --- | --- | --- | --- | --- | --- | --- | --- |
| 0(Subject) | 1 | 2 | 3 | 4 | 5 | 6 |  |  |
| Module Lengths (mm) | | | | | | |  |  |
| 0 | 100 | 200 | 400 | 800 | 1600 | 1600 |  |  |
| Module Configuration | | | | | | |  |  |
| X |  |  |  |  |  |  | 0 | 0 |
| X | X |  |  |  |  |  | 100 | 1 |
| X |  | X |  |  |  |  | 200 | 2 |
| X | X | X |  |  |  |  | 300 | 3 |
| X |  |  | X |  |  |  | 400 | 4 |
| X | X |  | X |  |  |  | 500 | 5 |
| X |  | X | X |  |  |  | 600 | 6 |
| X | X | X | X |  |  |  | 700 | 7 |
| X |  |  |  | X |  |  | 800 | 8 |
| X | X |  |  | X |  |  | 900 | 9 |
| X |  | X |  | X |  |  | 1000 | 10 |
| X | X | X |  | X |  |  | 1100 | 11 |
| X |  |  | X | X |  |  | 1200 | 12 |
| X | X |  | X | X |  |  | 1300 | 13 |
| X |  | X | X | X |  |  | 1400 | 14 |
| X | X | X | X | X |  |  | 1500 | 15 |
| X |  |  |  |  | X |  | 1600 | 16 |
| X | X |  |  |  | X |  | 1700 | 17 |
| X |  | X |  |  | X |  | 1800 | 18 |
| X | X | X |  |  | X |  | 1900 | 19 |
| X |  |  | X |  | X |  | 2000 | 20 |
| X | X |  | X |  | X |  | 2100 | 21 |
| X |  | X | X |  | X |  | 2200 | 22 |
| X | X | X | X |  | X |  | 2300 | 23 |
| X |  |  |  | X | X |  | 2400 | 24 |
| X | X |  |  | X | X |  | 2500 | 25 |
| X |  | X |  | X | X |  | 2600 | 26 |
| X | X | X |  | X | X |  | 2700 | 27 |
| X |  |  | X | X | X |  | 2800 | 28 |
| X | X |  | X | X | X |  | 2900 | 29 |
| X |  | X | X | X | X |  | 3000 | 30 |
| X | X | X | X | X | X |  | 3100 | 31 |
| X |  |  |  |  | X | X | 3200 | 32 |
| X | X |  |  |  | X | X | 3300 | 33 |
| X |  | X |  |  | X | X | 3400 | 34 |
| X | X | X |  |  | X | X | 3500 | 35 |
| X |  |  | X |  | X | X | 3600 | 36 |
| X | X |  | X |  | X | X | 3700 | 37 |
| X |  | X | X |  | X | X | 3800 | 38 |
| X | X | X | X |  | X | X | 3900 | 39 |
| X |  |  |  | X | X | X | 4000 | 40 |

A sample inlet thermohygrometer equipped with two probes is used to determine the temperature, relative humidity and water vapour concentration both upstream and downstream of the subject. One thermohygrometer probe denoted TRH-BG, is positioned behind the subject to measure the background temperature and RH is denoted TRH-BG while a second denoted TRH-S is located within the instrument module sample inlet. The inlet tubes for each of the aerosol instruments were brought into close proximity to TRH-S to ensure that sample thermohygrometer readings were representative of a common sample air parcel simultaneously entering all of the instruments. In order to eliminate potential impaction surfaces at the boundaries between the inlet orifices and to provide matched inlet velocities the individual instrument inlet tubes would ideally terminate in a tapered orifice such that sharp boundaries existed between all of these adjoining inlets. However, manufacturing limitations prevented this ideal from being achieved in the current iteration of the design. The inlet orifice cluster was integrated into single stainless steel conical orifice as shown in Fig D of the S1 File. The stainless steel cone was constructed from stacked closely fitting tapered concentric extension rings in order to provide some flexibility in matching of the inlet flow to the surrounding airflow. The inlet diameter can be varied through 4 steps from 63 to 177 mm by using these extensions and a range of sample inlet velocities can be thereby selected despite a fixed sample flow rate.

Conical Inlet

Thermohygrometer Probe


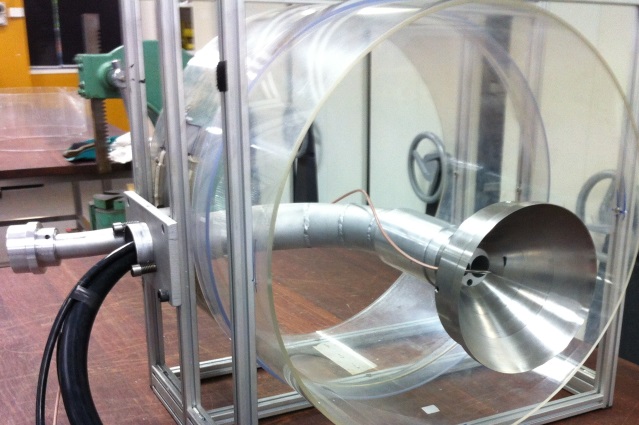


Aerosol Instrument sample tubes

ACI Mount

Fig D of the S1 File: TARDIS-tunnel Instrument module detail

The achievable sample inlet velocities are set out in Table B of the S1 File. The isokinetic performance of the probe was assessed by injecting indicator smoke and observing its passage past the probe. Super-isokinetic sample flow was revealed by the deflection of a passing smoke stream toward and into the cone while sub-isokinetic sampling deflects the stream away from and around the cone. The air velocity inside the TARDIS-tunnel was adjusted to match the inlet velocity, which eliminated all such deflection so that visually observed smoke streams did not deviate when passing or entering the cone.

Table B of the S1 File: Conical Inlet velocities achievable by varying the common inlet cone diameter when using the current TARDIS-tunnel instrument suite

|  | **Flow (L.min^-1^)** | **Inlet Diam (m)** | **Inlet Velocity (m.s^-1^)** | **Inlet Area (m^2^)** |
| --- | --- | --- | --- | --- |
| **TOTAL** | 74.1 | **0.177** | **0.05** | 0.025 |
|  | 74.1 | **0.125** | **0.1** | 0.012 |
|  | 74.1 | **0.089** | **0.2** | 0.006 |
|  | 74.1 | **0.063** | **0.4** | 0.003 |

Table C of the S1 File: Module and corresponding Rail Specification for the TARDIS-tunnel

| **Module Number** | **Type** | **Function** | **Duct Length (mm)** | **Rail allowance (mm)** | **Material** |
| --- | --- | --- | --- | --- | --- |
| -4 | SOH | Flex Overhang | 400 | 0 | FLEX |
| -3 | SOH | Inlet adaptor (to flexible duct) | 110 | 0 | Polycarbonate |
| -2 | SR | Inlet Straightener &  Humidity Sensor | 160 | 160 | Polycarbonate |
| -1 | SR | Subject Module | 500 | 500 | PERSPEX |
| 0 | SR | Post-subject spacer | 200 | 200 | PERSPEX |
| 1 | XR | eXtendeR | 100 | 100 | PERSPEX |
| 2 | XR | eXtendeR | 200 | 200 | PERSPEX |
| 3 | XR | eXtendeR | 400 | 400 | PERSPEX |
| 4 | XR | eXtendeR | 800 | 800 | PERSPEX |
| 5 | XR | eXtendeR | 1600 | 1600 | PERSPEX |
| 6 | XR | eXtendeR | 1600 | 1600 | PERSPEX |
| 7 | SR | Instrument | 220 | 220 | PERSPEX |
| 8 | SR | Outlet Straightener &  velocity | 200 | 200 | PERSPEX |
| 9 | SOH | Outlet adaptor to flexible duct | 110 | 0 | Polycarbonate |
| 10 | SOH | Flex Overhang | 400 | 0 | FLEX |
| System OverHang (SOH), SystemRail (SR), eXtenderRail (XR) | | | | | |

Table D of the S1 File: Dimensions of the sample tube orifices and inlet cone of the TARDIS-tunnel.

| **F_tidal_** | **VOL_produced_** | **F_UVAPS_** | **D_UVAPS in_** | **v_UVAPS in_** | **D_UVAPS iso_** | **F_OPC_** |
| --- | --- | --- | --- | --- | --- | --- |
| **(Lpm)** | **(L)** | **(Lpm)** | **(m)** | **(m/s)** | **(m)** | **(Lpm)** |
| 6.5 | 32.5 | 5 | 0.0189 | 0.297 | 0.0326 | 28.3 |
| **D_OPC in_** | **V_OPC in_** | **D_OPC iso_** | **F_SKC_** | **D_SKC in_** | **v_SKC in_** | **D_SKC iso_** |
| **(m)** | **(m/s)** | **(m)** | **(Lpm)** | **(m)** | **(m/s)** | **(m)** |
| 0.0094 | 6.8 | 0.077 | 12.5 | 0.0094 | 3.02 | 0.052 |
| **F_And_** | **D_And in_** | **v_And in_** | **D_And iso_** | **F_Total_** | **t_sample_** | **VOL_Needed_** |
| **(Lpm)** | **(m)** | **(m/s)** | **(m)** | **(Lpm)** | **(min)** | **(L)** |
| 28.3 | 0.0189 | 1.68 | 0.077494816 | 74.1 | 5 | 370.5 |
| **v_EDIS_** | **F_EDIS_** | **D^Cone^_tot iso_** | **SF_UVAPS_** | **SF_OPC_** | **SF_SKC_** | **SF_And_** |
| **(m/s)** | **(Lpm)** | **(mm)** | **WCFS^*^** | **WCFS^*^** | **WCFS^*^** | **WCFS^*^** |
| 0.1 | 1180 | 125 | 0.00424 | 0.0240 | 0.0106 | 0.0240 |
| ^*^WCFS = “Worst Case Fraction Sampled” | | | | | | |

## TARDIS-rotator:

A schematic diagram of the TARDIS-rotator is shown in Fig E of the S1 File below. The major aspects of the Rotator to be described are the rotating drum, inlet and outlet ports, sensor integration, rotation rate, aerosol handling, subject and instrument interfaces, subject connection time limitations.

Fig E of the S1 File: Schematic diagram of the TARDIS-rotator’s human subject and instrument interfaces.

## TARDIS-rotator Reservoir

Sealed bearings at each end permit rotation about the drum’s axis. The stationary inner part of each bearing has a central hole lined with O-rings, permitting tubes to be inserted so that air can be injected and extracted at either end of the drum. The overall system is airtight (tested to 1kPa) when the ends of these tubes are sealed.

The design was required to meet the following criteria:

1. Permit access for effective sterilisation.
2. Size and weight restriction to provide mobility.
3. Store exhaled breath under at room air temperature (T) and relative humidity (RH) (19°C≤T≤24°C, 30%≤RH≤70%).
4. Minimal dilution of the expired aerosol in order to reduce measurement uncertainty.
5. Age the diluted aerosol for periods of minutes to hours.
6. Permit extraction of aerosol to the ACI over a 5 minute interval as per the Cough Aerosol Sampling System (CASS) study[[2](#_ENREF_2)].
7. Store and hold a sufficient volume of cough and breath aerosol for a sufficient time to permit future studies of the hygroscopic properties of breath and cough aerosol using VH-TDMA analysis [[3](#_ENREF_3), [4](#_ENREF_4)].

Criterion 1 and 2 were met by using stainless steel (S/S) sheet (0.7 mm thick 304 S/S) for the drum walls to provide a rigid and readily cleaned surface without adding excessive weight. The end plates were to be easily removable for access when cleaning.

In order to ensure that the contents remained at room temperature the rotating drum was thermally coupled to the room air by enclosing the drum in a cabinet to shield it from radiant heat sources and ventilating the cabinet by using a small fan to draw room air through the enclosure, so that the thin walled drum was continuously bathed in a gentle flow of room air.

In order to estimate the minimum volume for the drum, such that the contents remain within the required room air RH range, the rise in RH inside the drum after a two minute breath injection was calculated as a function of drum volume. In order to allow for variations in breathing ventilation rate a conservative nominal value of 60% was chosen for the maximum RH in the drum. For this purpose the room air temperature was assumed to be 22°C and the RH was assumed to be 50%. The drum is assumed to have been filled with filtered room air under these conditions before breath injection. The calculation was based on the principal of matter conservation expressed in Equation C of the S1 File.

Based on the simulation described in section entitled RH limitations (Section 1.2.1.4.3), a nominal drum size of 400 L was chosen to allow approximately 200 seconds of breathing without excessively raising the drum RH. This condition was assumed to set the minimum achievable dilution without exceeding the requirement for maintaining room air conditions inside the drum.

In order to eliminate inaccessible aging intervals it was important to make the minimum aging interval for the TARDIS-rotator subsystem as close as possible to the maximum aging interval accessible by the TARDIS-tunnel. The maximum aging interval achievable using the 4 m long Tunnel depends on the air-flow speed used but is of the order of 1 minute. A significantly larger drum than the minimum size for maintaining room air conditions would unnecessarily dilute the aerosol sample so that longer sample extraction time would become necessary and this would reduce the minimum aging interval achievable.

The design was also required to permit ease of transport through doorways and elevators which limited the allowable dimensions to no wider than a standard doorway or 780 mm and no longer than 1500 mm to fit into elevators and allow manoeuvring around corners. In order to achieve the required drum volume while permitting transport through doorways and in elevators the drum dimensions provided in Table E of the S1 File were considered the most suitable.

The amount of aerosols collected by the ACI and hence the CFU count are limited by the sample extraction flow rate and time. Given the greatly reduced sample concentration envisaged due to dilution in the TARDIS-rotator, 5 minutes was determined to be the minimum extraction time. It follows that the minimum sample aging interval is 5 minutes if the intention is to be conservative by ensuring that all aerosol examined is *at least* 5 minutes old when collected. Assuming the sample extraction begins exactly 5 minutes after the last breath is injected into the drum the minimum age of any droplet nuclei collected is then 5 minutes. Alternatively, the age of the sample can be nominally assumed to be the median age in which case the minimum age is attained by commencing extraction immediately after injection has ceased. For a 2 minute injection this minimum median age is 3.5 minutes.

Previously published results for cough aerosol samples collected from CF patients infected with *P. aeruginosa* [[2](#_ENREF_2)] provide the range of colony forming units (CFU) collected without aging during 5 minutes of coughing with continuous simultaneous extraction using an ACI. These results can therefore be used to estimate the range of CFU counts likely to be observed in a comparable study using the TARDIS-rotator before the effect of aerosol aging reduces the CFU count. The ACI used to extract the aerosol samples for culturing draws 28.3 L min^-1^ so a 5 minute sample extraction equates to 141.5 L. Previous measurements using the Cough Aerosol Sampling System CASS[[5](#_ENREF_5)] showed that 5 minute voluntary coughing episodes by *Pseudomonas. aeruginosa* infected CF patients can be expected to yield a median total count of around 50 CFU [[2](#_ENREF_2)]. Assuming that this is representative, then based on 2 minutes of coughing (and assuming an average ventilation rate of 10.4 L min^-1^ [[6](#_ENREF_6)]) yields 20.8 L of breath. Assuming this exhaled breath simply displaces clean air from the 400 L chamber then this would yield a dilution factor of about 20 and 2-3 colonies for a 5 minute ACI sample assuming no loss of viable organisms due to aerosol aging. This was considered a challenging but workable result given that some patients exhibited CFU counts of 10000 or higher in a previous study[[2](#_ENREF_2)].

Allowing for dilution and assuming that the aerosol remains well mixed roughly 30% of the stored aerosol will be sampled by the ACI leaving sufficient aerosol for characterisation by the OPC and UV-APS.

Table E of the S1 File: TARDIS-rotator dimensions

| Diameter | 650 mm |
| --- | --- |
| Length | 1200 mm |
| Volume | 398 L |
| Rotational speed | 0.6-10 rpm |
| Axis Height above ground | 1150 mm |

## Reservoir rotation rate

The rotation of the drum limits gravitational settling losses, thereby permitting extended aging times which would otherwise require a much larger reservoir in which the sample would be highly diluted and where size dependant sedimentation would complicate interpretation of the resulting measurements. The aerosol remains suspended as a consequence of rotational momentum being transferred from the drum wall to the air inside so that the entire air mass rotates synchronously with the drum. With the entire air mass rotating slowly particles that would, in a stationary drum, fall in a straight line toward a fixed impact point on the drum wall now spend equal amounts of time falling alternately toward and away from the rotation axis. When viewed from its own centre of rotation in such a rotating cylinder, an individual particle will in fact move (in response to a combination of gravitational and viscous drag forces) in an expanding spiral that eventually intercepts the wall of the drum, removing that particle from the aerosol. The dynamics of this system were previously analysed to show that the fraction of particles retained (i.e. not subject to impaction or settling) after a period of storage is given by Equation A of the S1 File [[7](#_ENREF_7)].

Equation A of the S1 File: Fraction of particles retained according to Gruel et al. [[7](#_ENREF_7)]

$$\begin{matrix} \frac{N(t)}{N_{0}}=\left(\frac{R-r_{0}}{R}\right)^{2}e^{-kt}\text{, k}=2\tau\omega^{2},\text{ }r_{0}=\frac{\tau g}{\omega}\text{ } \\ \text{where} \\ \text{τ}=\text{relaxation time of the aerosol particle} \\ \text{g}=\text{acceleration due to gravity} \\ \omega=\text{angular velocity of the drum } \\ \text{N(t)}=\text{ number of particles in the drum at time t} \\ N_{0}=\text{ initial number of particles in the drum} \\ \text{R}=\text{ drum radius (m)} \\ \text{t}=\text{ aerosol aging time} \end{matrix}$$

The rotation rate for optimal aerosol retention within a given aging period was also determined according to Gruel el al. [[7](#_ENREF_7)]. For particles smaller than 10 µm in diameter the optimum rotation rate can be approximated by Equation B of the S1 File.

Equation B of the S1 File: Optimum rotation rate for a particle smaller than 10 µm from Gruel et al.[[7](#_ENREF_7)].

$$\omega=(g/2Rt)^{1/3}$$

For the chosen drum diameter of 650 mm the optimum rotation rate for particles in the size range 1-20 µm is in the range 1.5-1.7 rpm. In practice a rotation rate of 1.7 rpm was chosen because it was close to this target value and found to be result in a more stable and vibration free angular speed for the system that was constructed. According to Equation A of the S1 File, the fraction of particles retained after 1 hour in a drum this size and rotation rate ranges from 96% for 5 µm particles to 100% for 200 nm particles.

## Aerosol Handling Requirements

Typically the exhaled aerosol concentrations available from healthy subjects during breathing and coughing are very low in comparison to room aerosol concentrations. For example healthy subjects produce expired aerosol at concentrations of only 0.5cm^-3^ [[1](#_ENREF_1), [8](#_ENREF_8), [9](#_ENREF_9)], but ambient aerosol concentrations are typically far greater than this. Contamination with ambient aerosol would make it impossible to detect the signal of respiratory aerosols. Therefore, for the aerosol sample to remain free of contamination from ambient aerosol it is essential that the entire system including the subject’s RT, the drum and the associated plumbing are fully purged of any aerosol and that this entire system including the subject’s respiratory tract (RT) remain completely protected from the ambient aerosol throughout the experiment. The system and protocol must therefore provide for purging of the whole system including the subject’s RT and it must transition from this fully purged state through the sample collection, aging, and extraction processes without any opportunity for re-contamination.

## TARDIS-rotator Human Subject and Instrument Interfaces

The various elements of the system will now be identified. The schematic diagram in Fig E of the S1 File was introduced previously. It shows the preferred arrangement of the TARDIS-rotator subject interface and instrument interface. Minor changes to the configuration are possible and these will be discussed where relevant.

In order to understand the purpose of the various elements of the system the various states of the system during a typical experiment will be described. These states are summarised in Table F of the S1 File. The precise sequence of steps to be followed during a typical cough aerosol aging experiment will be discussed in detail in a later section describing the sampling protocol.

The subject and instrument interface systems provides plumbing necessary to allow the experiment to progress from purging the drum of particles to purging the subjects lungs of particles, capturing the subjects aerosol emissions, aging the sample and then analysing the sample without contamination by ambient aerosol.

The HEPA filter blower units HFB1 and HFB2 (3M HEPA Airmate Air Filter Unit) provide particle free air at 170 L min^-1^. Clean air from HFB1 is available to the subject when valve VI1 is open and the excess exits through a disposable bacterial filter (Pall BB50T).

**Drum Purge**: particle free airflow from both HEPA Blower 1 and HEPA Blower 2 is directed through the drum by moving HEPA blower 2 to Port P2, opening the two subject interface valves VI1 and VI2, blocking Port P1 and opening instrument interface valves VO1 and VO2. The concentration in the air leaving the drum is monitored continually using the OPC to identify when the concentration in the drum has fallen to zero.

**Drum Isolation:** Once particle free, the drum must be isolated to protect it from contamination. This is achieved by closing all flow paths to the drum by shutting the valves VI2, VO1, and VO2. Port P1 is then terminated by a disposable bacterial filter and P2 is opened. VI1 is left open to flush particles from all remaining flow paths of the subject interface and keep them particle free until the subject connects via a mouthpiece to P2. HFB2 is reconnected to the instrument interface as shown.

**Subject Airway Connection:** The subject sits wearing a nose clip and the chair height adjusted so that they can connect comfortably to the system via a disposable mouthpiece connected to P2 and will begin breathing particle free air supplied by HFB1.

**Subject Purge:** The subject continues breathing particle free air for at least 2 minutes to ensure that all particles originating from the ambient air have been flushed from the subject’s respiratory tract and replaced by HEPA filtered air. Typically the clearance of particles from the airways occurs in less than 1 minute however the extended breathing time allows the subject’s breathing to stabilise as they adapt to the mouthpiece, their comfort to be assessed and the experimental team to perform a final check of the equipment.

**Pre Capture:** With HFB2 reconnected to the Instrument interface and maintaining a particle free air path in the downstream plumbing and buffer volume, VO1 is opened.

**Capture:** VI1 and VI2 are simultaneously opened and the subject instructed to commence the planned expiratory manoeuvre (for example voluntary coughing). When the system is operated in the rebreathing (RB) configuration all air entering and leaving the subjects lungs is now sourced from the drum and subsequently expired back into the drum. The system is also capable of operating in a non-rebreathing (NRB) configuration in which the mouthpiece is connected to a T-piece fitted with a pair of one way valves to ensure that inhaled air is sourced from HFB1 but exhaled air is directed into the drum.

Regardless of which configuration is used, the air in the drum remains at atmospheric pressure because excess air displaced from or removed from the drum during breathing, is vented to the outdoor environment, or replaced with filtered air via the buffer volume. The air in this plumbing is continually purged by HFB2 so that during inhalation filtered air provided by HFB2 and stored in the buffer volume is drawn into the drum. The filtered air in the buffer volume ensures that instances of rapid inhalation momentarily exceeding the flow from HFB2 do not result in unfiltered room air reaching the drum and contaminating the sample. Instantaneous flow in the buffered vent line can be measured at 0.01 s temporal resolution by an optional ultrasonic flow meter (USF, Thor Medical Systems, THRUD medical flow meter). The spirotube flowmeter used here is designed to provide an unobstructed flow path and this ensures that no significant variation in the flow from HFB2 occurs during measurements. The USF was therefore able to be relocated to the vent line downstream of HFB2 with appropriate allowance for the resulting bias flow from HFB2 in the data analysis. The resulting addition of a bias flow of filtered room air from HFB2 to the breathing and cough induced flows when the USF is in this position results in a more stable temperature and humidity in the airflow through the device and a more stable flow measurement.

The NRB configuration was avoided due to concerns that large undried droplets moving at very high speeds would be lost during a cough through impaction on the valve membrane which must necessarily obstruct the path of the exhalation before the valve opens in response to pressure difference caused by the cough. The RB configuration has the advantage of doing away with this obstruction completely, but introduces the issue of CO_2_ build up as well as the potential for size selective aerosol losses due to deposition of previously expired droplet nuclei being inhaled and re-entering the lung. The issue of CO_2_ build up must be managed by limiting the cough aerosol accumulation time. The potential impact of size selective lung deposition losses will depend on the fraction of exhaled air which re-enters the lungs during a typical experiment. The maximum sample capture interval is also limited by the build-up of breath derived water vapour in the system. Each of these issues will be discussed in more detail in subsequent sections.

**Sample Isolation:** At the completion of the sample capture interval the valve VI2 is closed and the subject immediately disengages after which VO1 is also closed. At this point the sample is completely isolated in the drum. Note that VO2 remains closed.

**Sample Aging:** The sample is allowed to age in the rotating drum for a pre-determined interval.

**Sample Analysis:** Sample analysis typically involves an initial OPC scan followed by 5 minute extraction onto nutrient agar using the ACI then a second OPC scan followed by a UVAPS scan. An SMPS scan may also be performed if required. The bacterial filter is removed and P1 is closed using plug X. Valve VI1 is then opened so that the flow of particle free air from HFB1 can purge the sections of subject interface plumbing upstream of the closed vale VI2 and VI2 is then opened. The instrument to be used for analysis is then connected to port P3 before opening VO2 and simultaneously activating the instrument sample flow. The instrument draws air from the drum which is replaced by particle free air supplied by HFB1. In order to swap to a different instrument the instrument sample flow is deactivated at the same time as valve VO2 is closed and the instrument is disconnected from P3, and the next instrument connected before simultaneously activating the instrument flow and opening valve VO2.

Table F of the S1 File: System states and progression during a typical experiment

| **State** | **Ts (S)** | **tf (S)** | **Rotation** | **VI-1** | **VI-2** | **VO-1** | **VO-2** | **P1** | **P2** | **P3** |
| --- | --- | --- | --- | --- | --- | --- | --- | --- | --- | --- |
| **Drum Purge** | **0** | **900** | **On** | 1 | 1 | 1 | 1 | X | X | OPC |
| **Drum Isolate** | **900** | **910** | **On** | 1 | 0 | 0 | 0 | BF | Open | N/A |
| **Subject Connects** | **910** | **970** | **On** | 1 | 0 | 0 | 0 | BF | Subject | N/A |
| **Subject Purge** | **970** | **1090** | **On** | 1 | 0 | 0 | 0 | BF | Subject | N/A |
| **Pre Capture** | **1090** | **1100** | **On** | 1 | 0 | 1 | 0 | BF | Subject | N/A |
| **Capture** | **1100** | **1220** | **On** | 0 | 1 | 1 | 0 | BF | Subject | N/A |
| **Isolate sample** | **1220** | **1230** | **On** | 0 | 0 | 0 | 0 | BF | Subject | N/A |
| **Aging** | **1230** | **1230+Tage** | **On** | 0 | 0 | 0 | 0 | BF | Open | N/A |
| **Analysing** |  |  | **On** | 1 | 1 | 0 | 1 | X | Open | OPC/VS/UV-APS/SMPS |


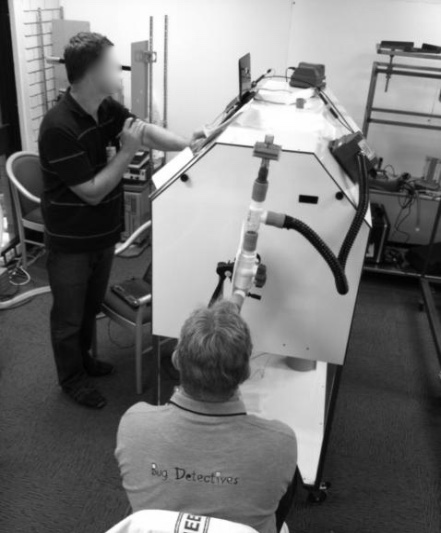

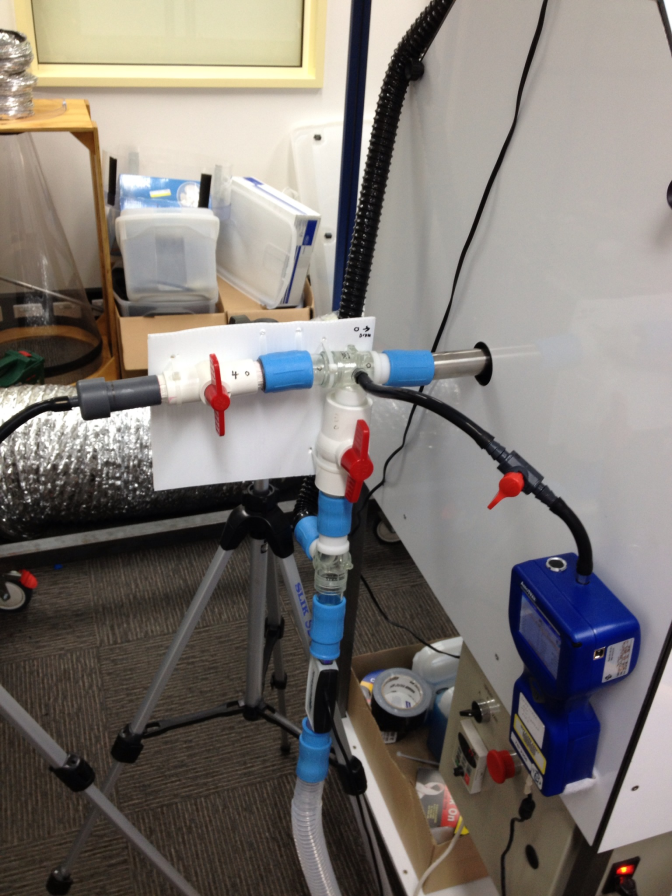


Fig F of the S1 File: A. Lung flushing prior to cough aerosol being injected into the TARDIS-rotator. B. Reservoir overflow path during exhalation indicated by red arrows with supplementary airflow indicated by blue arrows.

## Subject Connection Time Limitations

As discussed in the section above, the permissible sample collection interval is limited by the build- up of CO_2_ and water vapour in the drum.

## Air quality based limitations

The air quality in the TARDIS-rotator must be considered carefully because the breathing air supplied to the subject is sourced from a system in which ventilation with ambient air is limited. The O_2_ concentration and the CO_2_ concentration must be maintained within safe levels. Safe limits for both are presented in Table G of the S1 File along with typical ambient and exhaled breath concentrations.

The concentration of particles, H_2_O, O_2_ and CO_2_ in the drum can be predicted from the drum dimensions and the subject’s minute ventilation if the exhaled breath and ambient concentrations of the species in question are known and if the principal of conservation is assumed to hold as expressed in Equation C of the S1 File. Here the rate of change in concentration inside the drum is equal to the difference between the inflow and outflow of a species. Inflow occurs during both inhalation and exhalation. During inhalation the inflow consists of filtered ambient air entering the drum to replace air withdrawn by the subject. During exhalation the inflow consists of the breath exhaled by the subject. In both cases the average flow is equal to the subject’s minute ventilation (F). The total air flow rate into the drum is therefore equal to 2xF and this must be balanced by an equal out flow rate. The outflow consists of course of the air extracted from the drum during inhalation and displaced from the drum during patient exhalation and both components of this outflow carry particles, O_2_ and CO_2_ from the drum.

Equation C of the S1 File: conservation of matter (used to predict changes in H_2_O, O_2_ and CO_2_)

$$\begin{matrix} V\frac{dC(t)}{dt}=F(C_{A}+C_{B})-2FC(t) \\ \text{where} \\ \text{C(t)}=\text{ concentration of a species (eg Particles, H}_{2}\text{O, O}_{2}\text{ or CO}_{2}\text{) in the drum at time t} \\ \text{C}_{\text{A}}=\text{ concentration of the species in the ambient air} \\ \text{C}_{\text{B}}=\text{ concentration of the species in exhaled breath} \\ \text{F}=\text{ minute ventilation rate of the subject (L.min}^{\text{-1}}\text{)} \\ \text{V}=\text{volume of the drum (L)} \\ \text{C(t)}=\text{C}_{\text{A}}\text{ at t}=0 \end{matrix}$$

The required solution to Equation C of the S1 File must satisfy the initial condition C(0)=C_A_ because the drum is flushed with ambient air before the experiment begins. The required solution is therefore given by Equation D of the S1 File.

Equation D of the S1 File: Specific solution satisfying the initial condition C(t=0)=C_A_

$$C(t)=\frac{(C_{A}+C_{B})}{2}-\left(\frac{C_{B}-C_{A}}{2}\right)e^{-2Ft/V}$$

Table G of the S1 File: O_2_ and CO_2_ ambient air concentrations, exhaled breath concentrations and concentration limits for exposure at atmospheric pressure

|  | **Ambient** | **Typical Exhaled Breath** | **Minimum safe concentration** | **15 minute TWA limit** | **8 hour TWA limit** |
| --- | --- | --- | --- | --- | --- |
| O_2_ (ppm) | 2.09x10^5^ | 1.53 x10^5^ [[10](#_ENREF_10)] | 1.95 x10^5^ [[11](#_ENREF_11)] |  | - |
| CO_2_ (ppm) | 400 | 4.2 x10^4^ [[10](#_ENREF_10)] | - | 3.0 x10^4^ [[12](#_ENREF_12)] | 5 x10^3^ [[12](#_ENREF_12)] |

Fig G of the S1 File shows the decrease in [O_2_] and the increase in [CO_2_] when a patient is connected to the drum in the RB configuration. Note that the recommended 8 hour TWA limit for CO_2_ of 5000 ppm is exceeded long before the oxygen concentration falls below the recommended safe limit. The reduction in available O_2_ concentration in the air is therefore on first appearances, secondary to the CO_2_ concentration which can apparently reach toxic levels before oxygen depletion becomes a health issue. Note however that the subject is only breathing these concentrations for short periods and most of the time they are not attached to the drum. Also note that the permitted CO_2_ exposure should not be assessed on the basis of the concentration breathed momentarily but on the basis of integrated exposure over time. The exposure to elevated CO_2_ was therefore considered against the recommended cumulative time weighted exposure limit which is the relevant measure.

Fig G of the S1 File: Concentration of drum O_2_ and CO_2_ when the subject is re-breathing from the drum.

The cumulative time weighted average (TWA) exposure to CO_2_ in an 8 hour period increases with the total time in an eight hour interval during which air breathed by the subject is sourced from the drum. The predicted TWA exposure is shown in Fig H of the S1 File along with the thresholds for allowable 8 hour TWA exposure and the toxicity threshold. In order to build a safety margin into the model a conservatively high minute volume of 30 L min^-1^ was assumed and the upper limit for exhaled CO_2_ concentration of 6.0x10^4^[[13](#_ENREF_13)] was used in place of the typical exhaled breath concentration given in Table G of the S1 File.

Fig H of the S1 File: Eight hour TWA Exposure to CO_2_ as a function of cumulative drum breathing time in an eight hour day. Also shown are the eight hour TWA exposure limits (orange dashed horizontal line) and toxic threshold (solid red horizontal line).

Given these finding the O_2_ concentration was finally determined to be the more important consideration given that the concentration falls below the acceptable limit after 4.5 minutes while the exceedance of CO_2_ 8hr TWA limit takes far longer. The maximum allowed drum connection time in any single test is therefore defined as 4.5 minutes per 8 hour interval, however in practice this was reduced to 2 minutes to provide an added safety margin.

The integrated TWA CO_2_ exposure falls quickly back to the ambient baseline value when the subject disengages from the drum and goes back to breathing room air and no cumulative measure of oxygen restriction is relevant at these levels so subjects can be permitted to perform repeated testing provided that a suitable recovery period is permitted. Five instances of drum exposure of two minute duration each were deemed acceptable with appropriate monitoring by medical personal.

## Loss of droplet nuclei due to re-inhalation

The potential magnitude of the effects of exhaled droplet nuclei being lost through particle re-deposition in the RT when re-breathing exhaled aerosol can be examined by calculating the fraction of exhaled breath which is re-inhaled during a typical cough aerosol capture experiment using the TARDIS-rotator.

The expected increase in the fraction of nuclei re-inhaled with capture duration is shown in Fig I of the S1 File. During a 2 minute capture experiment using the RB configuration less than 10% of the exhaled droplet nuclei will be re-inhaled. Therefore a maximum of 10% loss to the subjects RT can be expected. Respiratory tract deposition occurs primarily through impaction and gravitational sedimentation for nuclei larger than 1 µm and through diffusion deposition for nuclei smaller than 0.1 µm[[14](#_ENREF_14)] so that probability of an inhaled nucleus being deposited varies substantially with size. This size dependence loss mechanism will therefore introduce a discrepancy of up to 10% in the measured size distribution.

Fig I of the S1 File: Cumulative fraction of exhaled breath, which has been re-inhaled at some point during the experiment for three subject ventilation rates. MV refers to minute volume in litres.

## RH limitations

In order to assess the likely effect of exhaled moisture, the rise in RH inside the drum after a two minute breath injection was calculated as a function of drum volume in order to estimate the minimum volume for the drum in order that the contents remain within the required room air RH range. In order to allow for variations in breathing ventilation rate, a conservative nominal value of 60% was chosen for the maximum RH in the drum. For this purpose the room air temperature was assumed to be 22 °C and the RH was assumed to be 50%. The drum is assumed to have been filled with filtered room air under these conditions before breath injection. The calculation was performed using Equation C of the S1 File.

CF patients were assumed to have a mean resting ventilation rate of 10.4 L min^-1^ [[6](#_ENREF_6)]. Breath was assumed to be saturated with water vapour at body temperature prior to exiting the RT. The breath was also assumed to be injected into the drum without any water loss through condensation. The drum was assumed to be held at room temperature via the thermal coupling of the drum to the room air. The RH growth inside the drum when a patient inhales from and exhales into the drum at an ambient T and RH of 22°C and 50% was calculated and is shown in Fig J of the S1 File. The growth in RH is also shown for a ventilation rate 75% higher than the mean for resting CF patients to allow for increased ventilation during coughing.

Fig J of the S1 File: RH increase with time for a patient breathing from the drum.

## RH and T sensor Integration

RH and T inside and outside the drum are measured at regular intervals using a pair of thermohygrometer probes (Rotronic HC2-C04, Rotronic Instrument Corp., Huntington, NY, USA) connected to a multichannel handset (Rotronic HygroPalm 2). Both signals are also displayed and logged on a laptop computer mounted on the cabinet wall. The internal probe was inserted through a neoprene grommet in the wall of the drum rotation bearing adjacent to the sample extraction tube fitting at the sample extraction end of the Rotator reservoir. The ambient RH and T in the room outside the TARDIS-rotator cabinet were measured using the second probe which was mounted on the outer cabinet.

## TARDIS-tunnel sample collection and aging procedures

In order to minimise the potential for cross infection, only one subject is tested on any given day and the TARDIS-tunnel is thoroughly disinfected at the end of each testing session. Each testing day commences with a radial swab sample taken from the interior surface of the clean Tunnel directly in front of the subject position. The swab sample is a disinfection quality assurance measure intended to monitor the adequacy of the Tunnel disinfection measures by verifying the absence of target organism in the system.

Each subject sample is preceded by the collection of a sample blank designed to provide assurance of the absence of airborne bacteria in the particle free Tunnel airflow such as through the entry of room air into the system or suspension of residue from the walls of the Tunnel.

## TARDIS-tunnel subject procedure.

The subject sample collection procedure collects a typically 5 minute ACI sample simultaneously with OPC, APS and thermohygrometer measurements while the subject is performing a respiratory manoeuvre such as breathing, speaking or coughing in the TARDIS-tunnel.

In order to position the subject in the Tunnel the subject is first seated below the subject portal in the motorised chair. The chair is then raised and the subject’s head enters the Tunnel through a portal on the underside of the subject module. Closure of the subject entry portal is necessary during sampling in order to minimise air loss from the Tunnel and maintain the positive pressure necessary to prevent the entry of room air through the subject entry portal. This closure is not required to be a perfect seal because of the overpressure from additional air entering the system via the adjustable butterfly valve in the HEPA blower unit (see Fig B of the S1 File). A partial seal is nevertheless required and this is provided by a cylindrical cloth shroud attached by Velcro^TM^ around the lip of the portal. The shroud is weighted at its base which allows it to wrap loosely about the subject’s upper torso without ‘bunching’ and seals the opening. If required, the position of the instrument cluster inlet probe can then be adjusted to align it with the subject’s mouth.

The subject is asked to sit quietly until given a signal to begin coughing. The aerosol concentration is monitored visually to verify that the concentration falls as residual room air particles are flushed from the patient’s lungs and the Tunnel. After the concentration has stabilised a timer is activated, and the patient observes quiet breathing while OPC, APS and thermohygrometer readings are logged to record the breathing aerosol size distribution as well as the RH and T readings required for dilution correction. OPC measurements in real time are used to verify that no room air particles are present before each test, the subjects’ lungs are free of residual room air, and no contamination of aerosols by the ingress of room air is occurring. In order to proceed with the experiment the total particle concentration in the Tunnel is required to be below 0.01 cm^-3^ prior to the subject commencing the manoeuvre, which is similar to an ISO 4 cleanroom and typically 1/10000 of the room air concentration.

The subject is then asked to commence the designated expiratory manoeuvre and the ACI is then activated. Typically the manoeuvre is continued for 5 minutes before the subject is instructed to cease the manoeuvre and resume quiet breathing and the ACI pump is stopped. As a quality control measure the operator then verifies that the concentration in the Tunnel falls back to the level previously observed for quiet breathing before the instrument logging is stopped and the subject is lowered from the Tunnel. The ACI is then removed from the instrument module for processing.

## TARDIS-tunnel blank procedure

The TARDIS-tunnel blank procedure is essentially identical to the subject procedure but without the subject present in the Tunnel. The subject entry portal is normally closed by the subject’s upper torso wrapped in the subject shroud. During the blank procedure the portal was instead closed by tying off the shroud. The aerosol concentration in the Tunnel is monitored via the OPC, and the ACI sample commenced once the concentration has stabilised at or near zero.

## **TARDIS-rotator sample collection and aging procedures**

As for the TARDIS-tunnel system only one subject is tested on any given day, and the Rotator and fittings are thoroughly disinfected at the end of each testing session by manual cleaning and autoclave respectively. Each testing day commences with a radial swab sample taken from the interior surface of the clean Rotator drum, directly in front of the inlet port, as a disinfection quality assurance measure verifying the absence of target organisms in the system. This swab is intended to monitor the adequacy of the reservoir disinfection measures. Each subject sample is preceded by the collection of a sample blank designed to provide assurance of the absence of airborne bacteria in the particle free Tunnel airflow such as through the entry of room air into the system.

## TARDIS-rotator subject procedure.

The full step-by-step TARDIS-rotator subject experimental procedure is shown in Table H of the S1 File. The subject procedure is designed to step sequentially through each of the system states defined in Table F of the S1 File and more fully described in the text preceding that table. Thermohygrometer readings of the internal and external RH and temperature are continually logged throughout the subject procedure, in part to provide information about the sample aging environment but also to provide the information required to calculate the breath dilution factor for the sample. The procedure begins with a drum purge to replace the air inside with HEPA filtered particle free air while monitoring the fall in concentration via the OPC. Following purging the drum is isolated to prevent contamination. Using a new sterile mouthpiece, fitted with a nose peg and sitting comfortably at the subject interface (schematic in Fig E of the S1 File and photograph in Fig F of the S1 File) the subject connects to the system via the mouthpiece. The subject purge state supplies HEPA filtered particle free air for the subject to breathe. The subject is instructed to breathe quietly. Quiet breathing continues for 2 minutes to clear the subjects lungs of particles previously inhaled with the room air. After 2 minutes, the system is placed in the pre capture state by connecting the drum to the particle free exhaust system. USF exhaust flow rate logging is initiated at this point to record all inflows and outflows from the drum, providing a measure of the subject ventilation rate during the manoeuvre as well as a second means of assessing the breath dilution factor. The capture state is then entered by diverting the mouthpiece to the drum. At this point the subject is instructed to commence the designated respiratory manoeuvre and, in the RB configuration, all breath is now sourced from and delivered to the drum with displaced air being released via the exhaust vent system and inhaled air being replaced by particle free air which is also continually injected into the exhaust system. At the end of the desired capture time the isolation valves are closed to isolate the sample and the subject is simultaneously instructed to cease the respiratory manoeuvre and disengage from the mouthpiece. The system remains isolated for the desired aging period. At the end of the aging period the system is switched to the sample analysis state.

Sample analysis typically begins with a brief (1 min) size distribution scan using the OPC. This is followed by extraction of an ACI sample (typically 5 min). The ACI is handed over for processing as a second size distribution scan is commenced using the OPC. The two size distribution scans recoded using the OPC are used to assess the change in concentration due to the ACI sample extraction as all air withdrawn from the system is replaced by HEPA filtered air and this results in further sample dilution. Additional size distribution scans may then be performed using the UV-APS and SMPS if required.

## TARDIS-rotator blank procedure.

The full step by step TARDIS-rotator blank sample collection procedure is shown in Table I of the S1 File. The Rotator blank procedure is identical to the subject procedure but as it does not involve a subject connecting to the system it necessarily bypasses steps 3, 4, 5, 6 and 7. Thus the system is simply purged and thereby filled with HEPA filtered air which is then isolated. The system is then put through the remaining analysis steps as per the subject procedure. The ACI samples are used for quality assurance to verify the absence of culturable bacteria in the system and thereby eliminate the possibility of outside contamination of the sample.

## Relative Humidity and Temperature in the TARDIS

## RH and T in the TARDIS-tunnel

A

B

C

Fig K of the S1 File: Example of TARDIS-tunnel experiment behaviour in the lead up to and during the cough manoeuvre of a typical experiment. Panel A: Temperature, Panel B: Relative Humidity, Panel C: Water vapour concentration and sample dilution factor. In the legend of each panel “up” refers to the upstream measurement of the particle free background air flow and “do” refers to a downstream measurement of the sample entering the instrument cluster probe and containing a mixture of background air and exhaled, breath air.

## RH and T in the TARDIS-rotator

The time series of the temperature and RH inside and outside the TARDIS-rotator in the lead up to and during the CRBE45 experiment cough manoeuvre is shown in Fig L of the S1 File panel A and panel B respectively.

The mean and standard deviation of the RH, temperature (T) and sample dilution factor (DF) in cough samples across the three experiments during the TARDIS-tunnel experiments were RH: 59.2(5.1)%, T: 22.8(1.2)°C, DF: 28.1(9.5) while for the TARDIS-rotator experiments they were RH: 63.9(7.2)%, T: 22.7(1.2)°C, DF 10.8(2.9).

A

B

C

D

**D**

Fig L of the S1 File: Example of a TARDIS-rotator experiment behaviour in the lead up to and during the S245 Experiment cough manoeuvre. Panel A: Temperature, Panel B: Relative Humidity, Panel C: Water vapour concentration and sample dilution factor as calculated from the water vapour concentration change, Panel D: Subject ventilation rates obtained from the drum exhaust vent line flow by subtracting the bias flow. The voluntary coughing manoeuvre phase is defined by vertical green lines. Red lines define a bias flow measurement interval during the period when the drum was isolated prior to opening VI2, green lines indicate flows at the commencement of the coughing manoeuvre. Green curve labelled D in panel D represents the dilution factor calculated from the flow measurements.

## Dilution Correction

The aerosol concentrations measured in exhaled breath which has been diluted with particle free air can be corrected to obtain the original concentration at the mouth if the appropriate DF is known.

Dilution refers to a change in the concentration of an airborne exhaled droplet nuclei caused by a change in the ratio of breath to filtered room air. Dilution affects the concentration of all airborne breath constituents including droplet nuclei, CO_2_ and water vapour but in the case of CO_2_ and H_2_O the presence of these constituents in the dilution air must also be taken into account. Differences in the design and operation of the TARDIS-tunnel and Rotator, result in differences in sample dilution. The extent of variation in dilution is also altered in each by subject and experimental parameter changes. Subject induced dilution variance occurs with changes in the subject’s ventilation rate. Experimentally induced variance in the dilution also occurs in the TARDIS-tunnel through changes in the spatial distribution of the exhaled breath plume relative to the surrounding Tunnel. This affects the faction of the breath plume captured by the cone, and hence the ratio of breath plume to filtered air entering the cone. The spatial distribution is affected by changes in the strength of the exhalation as well as by head and facial movements of the subject which alter the shape and direction of the cough jet. In principle it is possible to correct the viable size distribution for dilution if the breath DF is known. All dilution effecting the droplet nuclei concentration can also be corrected if the breath DF is known.

The DF is identical for each airborne species provided that losses do not occur and any addition of the species in the dilution air is accounted for. Water vapour can be used to assess the relevant DF for particles provided that loss of moisture through condensation onto surfaces does not occur and the presence of water vapour in the dilution air is accounted for. The DF applicable to water vapour derived from that subject’s respiratory tract can be determined using Equation E of the S1 File and Equation F of the S1 File from measurements of RH and T in the diluted sample and in the dilution (background) air.

Equation E of the S1 File: Calculating the sample dilution factor from water vapour concentration measurements

$$\begin{matrix} DF=\frac{C_{RT}-C_{BG}}{C_{s}-C_{BG}} \\ \text{where} \\ C_{RT}=\text{ water vapor concentration in the respiratory tract} \\ C_{s}=\text{ water vapor concentration in the sample} \\ C_{BG}=\text{ water vapor concentration in background air } \end{matrix}$$

Equation F of the S1 File: Calculating the water vapour concentration from the measured RH and temperature

$$\begin{matrix} C=\frac{RH}{100}\left(\frac{p_{sat}MW_{H_{2}O}}{RT}\right) \\ RH=\text{ relative humidity (\%)} \\ p_{sat}(T)\text{ }=\text{ saturation vapour pressure of water at T} \\ MW_{H_{2}O}\text{ }=\text{ molecular weight of water} \\ R\text{ }=\text{ gas constant} \\ T=\text{ temperature (K)} \end{matrix}$$

The use of a tracer gas to estimate dilution generally relies on plume dispersion occurring primarily through homogenous mixing. In reality the processes causing the dispersion can occur at different rates for gasses and particles. This issue includes the different gravitational settling velocities of the various particle sizes observed. Nevertheless the problem is in this application, effectively offset by the fact that during sampling, the location of the volunteers mouth and the direction of the expired jet vary continually, so the probe in effect takes an average at many random locations over an area with a diameter of the order of 50 mm, based on observed movements of the mouth relative to the probe. The expected gravitational settling velocity for unit density particles in the largest particle size mode (7 µm) observed during the experiment is 1.5 mm s^-1^. The estimated penetration efficiency for 7 µm particles in a horizontal tube with the same dimensions as the EDIS APS probe tube under the similar flow conditions[[15](#_ENREF_15)] is 92% so that losses in the probe tube can also be neglected.

## Dilution in the TARDIS-tunnel

Panel C of Fig K of the S1 File shows the water vapour concentration upwind of the subject and in the entrance to the instrument cluster inlet cone as calculated from the measured RH and T according to Equation F of the S1 File. The dilution factor is shown as calculated according to Equation E of the S1 File. This calculation assumes that the breath is saturated with water vapour at a temperature of 31.5 °C inside the respiratory tract where the aerosol is generated. This assumption corresponds to a water vapour concentration which was 33.24 g m^-3^ in the aerosol prior to exiting the RT. The dilution factor for the sampled aerosol with respect to its initial state at the site of production was taken to be the average value during sample extraction.

## Dilution in the TARDIS-rotator

Panel C of Fig L of the S1 File shows the water vapour concentration inside and outside the drum as calculated from the measured RH and T according to Equation F of the S1 File. The dilution factor is again shown as calculated according to Equation E of the S1 File. Unlike the TARDIS-tunnel, the dilution factor in the Rotator increases during sample extraction so the dilution factor for the sampled aerosol with respect to its initial state at the site of production is taken to be the midpoint between the value of D immediately prior to sample extraction and immediately after sample extraction.

## Verification of the water vapour based dilution factor using flow measurements

In order to validate the approach to dilution measurement, a dilution factor was derived simultaneously by an alternate method based on the flows into and out of the drum.

Note that with the subject connected and wearing a nose clip and with VI2 and VO2 both closed, the TARDIS-rotator and patient together form a closed system apart from the single flow path to the exhaust vent line. Therefore after correcting for the positive offset due to the bias flow from HFB2, the flows in the drum exhaust vent line equate to the subject’s breathing flow. The offset corrected exhaust flow during the subject lung purge and subsequent 2 minute cough manoeuvre are presented in Panel D of Fig L of the S1 File. During the cough manoeuvre the subject was connected to the drum so the flow within the green boundary represents the breathing flow.

The average of the cumulative volume inhaled per unit time and cumulative volume exhaled per unit time is also shown in the graph. This is a measure of the breathing ventilation rate. In the test represented in the graph the subject’s breathing ventilation rate increased from an initial value of 17 L min^-1^ at the start of the manoeuvre to a final value of 20 L min^-1^ at its completion the cough manoeuvre.

The DF can be calculated from the breathing ventilation rate according to Equation G of the S1 File. The behaviour of the dilution factor is shown by the green curve in the aforementioned figure. At the completion of the manoeuvre the dilution factor was 10.1. The DF obtained using water vapour ranged from 8.6 to 11.5 at the completion of the manoeuvre. The agreement shows that the DFs calculated using the water vapour based technique is accurate to within 15% for TARDIS-rotator measurements. A comparable level of accuracy was assumed for the TARDIS-tunnel measurements.

Equation G of the S1 File: Calculation of the breath dilution factor (DF) from the breathing ventilation rate.

$$\begin{matrix} DF=\frac{1}{\left(1-e^{-\left(\frac{BVR}{V}(t-t_{0}\right)}\right)} \\ \text{where} \\ BVR=\text{ Breathing ventilation rate} \\ V=\text{ Drum volume } \\ \text{t}_{0}\text{ }=\text{ time at which the coughing maneuvre commenced} \end{matrix}$$

| **DURATION - Subject Procedure** | | | |
| --- | --- | --- | --- |
| **Stage** | **Step** | **Procedure** | **Additional tasks** |
| 1. Flush | 1 | BLOWER **WITHOUT T** TO INLET |  |
|  | 2 | Open V1, V3 & V5 |  |
|  | 3 | BLOWER ON - check flow |  |
|  | 4 | OPC1 to V4 |  |
| Flush Start | 5 | OPC 1 & 2 START | Row A: Record time |
|  | 6 | OPC 1&2 to stabilise (15 min) | Row B: Record time |
|  | 7 | OPC 1&2 Stop |  |
|  | 8 | Close V5, V4 & V1 |  |
|  | 9 | BLOWER OFF |  |
|  | 10 | Close V3 |  |
|  | 11 | BLOWER **WITH T** TO EXIT with buffer tube |  |
|  | 12 | Check RPM |  |
|  | 13 | BLOWER ON check flow |  |
|  | 14 | Open V2 |  |
|  | 15 | Connect Mouth piece |  |
|  | 16 | USF on & check logger |  |
| Patient setup | 17 | Position patient |  |
|  | 18 | Apply noseclip to patient |  |
|  | 19 | Apply mouthpiece to patient |  |
| Breathing | 20 | Wait for 2 minutes lung purge | Row C: Record breathing start and required end time |
|  | 21 | Open V3 |  |
|  | 22 | Open V1 |  |
|  | 23 | Close V2 |  |
| Coughing | 24 | Patient cough 2 min | Row D: Record cough start & required end time |
| Ageing | 25 | Close 1 |  |
|  | 26 | Close 3 | Row E: Record aging start and required end time |
|  | 27 | BLOWER **WITH T** TO INLET - check flow |  |
|  | 28 | USF Stop |  |
|  | 29 | Wait for remainder of ageing period |  |
| Pre extraction OPC | 30 | Open V1 V4 & V5 |  |
|  | 31 | OPC 1&2 START |  |
|  | 32 | Wait 60 seconds (6 scans) | Row G: verify OPC stabilisation and record time |
|  | 33 | OPC 1&2 STOP |  |
|  | 34 | Close V4 & V5 |  |
| Extraction | 35 | Record ACI Serial Number |  |
|  | 36 | Attach ACI |  |
|  | 37 | Attach ACI pump tube |  |
|  | 38 | Open 4 |  |
|  | 39 | ACI on | Row H: record ACI start and required end time |
|  | 40 | Wait 5 minutes |  |
|  | 41 | ACI Off |  |
|  | 42 | Close 4 |  |
|  | 43 | Remove ACI and attach OPC1 to V4 |  |
| Post extraction OPC | 44 | Open V4 & V5 |  |
|  | 45 | OPC 1 & 2 START | Row J: record time |
|  | 46 | Wait 60 seconds (6 scans) | Row K: verify OPC stabilisation and record time |
|  | 47 | OPC 1 STOP |  |
|  | 48 | Close V4 | Row L: Record number of coughs |
|  | 49 | Attach APS |  |
|  | 50 | Open V4 |  |
|  | 51 | Wait 5 minutes |  |
|  | 52 | OPC 2 Stop |  |
|  | 53 | Close V4 & V5 |  |
|  | 54 | Remove APS |  |
|  | 55 | Open 3 |  |

Table H of the S1 File: Subject procedure for TARDIS-rotator cough experiments.

| **DURATION - Blank Procedure** | | | |
| --- | --- | --- | --- |
| **Stage** | **Step** | **Procedure** | **Additional tasks** |
| Flush | 1 | BLOWER **WITHOUT T** TO INLET |  |
|  | 2 | Block Filter & Open 2 |  |
|  | 3 | Open V3, V4 & V5 |  |
|  | 4 | Long Vent Tube |  |
|  | 5 | BLOWER ON - Check Flow |  |
|  | 6 | OPC 1 to V4 |  |
|  | 7 | OPC 1 & 2 START | Row A: Record start time |
| Pre Extraction QC | 8 | Continue flushing until OPC 1 & 2 concentration falls to zero |  |
|  | 9 | OPC 1 & 2 STOP | Row B: Record finish time |
| Isolate | 10 | Close V5, V4, V1 & V2 |  |
| Extract Setup | 11 | Unblock Filter |  |
|  | 12 | BLOWER OFF |  |
|  | 13 | **Close V3** |  |
|  | 14 | BLOWER **WITH T** TO **INLET** |  |
|  | 15 | BLOWER ON check flow |  |
|  | 16 | Record ACI Serial Number |  |
|  | 17 | Attach ACI |  |
|  | 18 | Attach ACI pump tube |  |
|  | 19 | Open V1 & V4 |  |
| Extract | 20 | ACI on | Row C: record start time |
|  | 21 | wait 5 minutes |  |
|  | 22 | ACI Off | Row D: record end time |
|  | 23 | Close V4 |  |
|  | 24 | Remove ACI |  |
|  | 25 | OPC 1 to V4 |  |
| Post Extraction QC | 26 | Open V4 & V5 |  |
|  | 27 | OPC 1 & 2 Start | Row E: Record start time |
|  | 28 | Wait 60 seconds (6 scans) |  |
|  | 29 | OPC 1 & 2 Stop | Row F: Record stop time |
| Isolate | 30 | Close V5 & V4 |  |
|  | 31 | Close V1 & V2 |  |

Table I of the S1 File: Blank procedure for TARDIS-rotator cough experiments

## Supplement to the results

Fig M of the S1 File: Average concentration in each size channel of the OPC and ACI recorded at distances of 1m (10 seconds) 2m (20 s) and 4m (40 s) from subjects S1 and S2. Diameters represent the lower boundaries of each OPC or ACI size channel.

Fig N of the S1 File: Average concentration in each size channel of the OPC and ACI recorded after durations of 300 s (5 min), 900 s (15 min) and 2700 s (45 min) for subjects S1 and S2. Diameters represent the lower boundaries of each OPC or ACI size channel.

# References

1. Morawska L, Johnson GR, Ristovski ZD, Hargreaves M, Mengersen K, Corbett S, et al. Size distribution and sites of origin of droplets expelled from the human respiratory tract during expiratory activities. Journal of Aerosol Science. 2009;40(3):256-69. doi: DOI 10.1016/j.jaerosci.2008.11.002. PubMed PMID: ISI:000264353400006.

2. Wainwright CE, France MW, O'Rourke P, Anuj S, Kidd TJ, Nissen MD, et al. Cough-generated aerosols of *Pseudomonas aeruginosa* and other Gram-negative bacteria from patients with cystic fibrosis. Thorax. 2009;64(11):926-31. doi: DOI 10.1136/thx.2008.112466. PubMed PMID: ISI:000271265100004.

3. Johnson GR, Ristovski Z, Morawska L. Method for measuring the hygroscopic behaviour of lower volatility fractions in an internally mixed aerosol. Journal of Aerosol Science. 2004;35(4):443-55.

4. Johnson GR, Ristovski ZD, D'Anna B, Morawska L. Hygroscopic behavior of partially volatilized coastal marine aerosols using the volatilization and humidification tandem differential mobility analyzer technique. Journal of Geophysical Research-Atmospheres. 2005;110(D20):-. doi: Artn D20203

Doi 10.1029/2004jd005657. PubMed PMID: ISI:000233106300001.

5. Fennelly KP, Martyny JW, Fulton KE, Orme IM, Cave DM, Heifets LB. Cough-generated aerosols of Mycobacterium tuberculosis - A new method to study infectiousness. American Journal of Respiratory and Critical Care Medicine. 2004;169(5):604-9. doi: DOI 10.1164/rccm.200308-1101OC. PubMed PMID: ISI:000189249300014.

6. Bell SC, Saunders MJ, Elborn JS, Shale DJ. Resting energy expenditure and oxygen cost of breathing in patients with cystic fibrosis. Thorax. 1996;51(2):126-31. Epub 1996/02/01. PubMed PMID: 8711641; PubMed Central PMCID: PMC473014.

7. Gruel RL, Reid CR, Allemann RT. The optimum rate of drum rotation for aerosol aging. Journal of Aerosol Science. 1987;18(1):17-22. doi: 10.1016/0021-8502(87)90004-8.

8. Johnson GR, Morawska L. The Mechanism of Breath Aerosol Formation. Journal of Aerosol Medicine and Pulmonary Drug Delivery. 2009;22(3):229-37. doi: DOI 10.1089/jamp.2008.0720. PubMed PMID: ISI:000269794700002.

9. Johnson GR, Morawska L, Ristovski ZD, Hargreaves M, Mengersen K, Chao CYH, et al. Modality of human expired aerosol size distributions. Journal of Aerosol Science. 2011;42(12):839-51. doi: 10.1016/j.jaerosci.2011.07.009.

10. Altman PL, Dittmer DS. Summary of values useful in pulmonary physiology: man. Bethesda, Maryland Federation of American Societies for Experimental Biology; 1971.

11. Safe_Work_Australia. Confined Spaces Code of Practice. Canberra, ACT Australia2011.

12. Safe_Work_Australia. Workplace Exposure Standards For Airborne Contaminants. Canberra, ACT Australia2011.

13. Smith D, Pysanenko A, Spanel P. The quantification of carbon dioxide in humid air and exhaled breath by selected ion flow tube mass spectrometry. Rapid Commun Mass Spectrom. 2009;23(10):1419-25. Epub 2009/04/07. doi: 10.1002/rcm.4016 [doi]. PubMed PMID: 19347971.

14. Heyder J. Deposition of Inhaled Particles in the Human Respiratory Tract and Consequences for Regional Targeting in Respiratory Drug Delivery. Proceedings of the American Thoracic Society. 2004;1(4):315-20. doi: 10.1513/pats.200409-046TA.

15. Baron PA, Willeke K. Aerosol Measurement: Principles, Techniques, and Applications. Second Edition ed. New York: John Wiley & Sons; 2001. 1131 p.
